# Plasma protein *N-*glycosylation is associated with cardiovascular disease, nephropathy, and retinopathy in type 2 diabetes

**DOI:** 10.1136/bmjdrc-2021-002345

**Published:** 2021-10-13

**Authors:** Elham Memarian, Leen M 't Hart, Roderick C Slieker, Roosmarijn F L Lemmers, Amber A van der Heijden, Femke Rutters, Giel Nijpels, Emma Schoep, Aloysius G Lieverse, Eric J G Sijbrands, Manfred Wuhrer, Mandy van Hoek, Viktoria Dotz

**Affiliations:** 1 Center for Proteomics and Metabolomics, Leiden University Medical Center, Leiden, The Netherlands; 2 Genos Glycoscience Research Laboratory, Zagreb, Croatia; 3 Department of Cell and Chemical Biology, Leiden University Medical Center, Leiden, The Netherlands; 4 Department of Epidemiology and Biostatistics, Amsterdam University Medical Center, location VUmc, Amsterdam, The Netherlands; 5 Department of Biomedical Data Sciences, Section Molecular Epidemiology, Leiden University Medical Center, Leiden, The Netherlands; 6 Department of Internal Medicine, Erasmus Medical Center, University Medical Center, Rotterdam, The Netherlands; 7 Amsterdam UMC, Vrije Universiteit Amsterdam, Department of General Practice Medicine, Amsterdam Public Health Research Institute, Amsterdam, The Netherlands; 8 Amsterdam UMC, Vrije Universiteit Amsterdam, Department of Epidemiology and Data Science, Amsterdam Public Health Research Institute, Amsterdam, The Netherlands; 9 Department of Internal Medicine, Maxima Medical Center, Eindhoven, The Netherlands

**Keywords:** biomarkers, diabetes complications, glycosylation

## Abstract

**Introduction:**

Although associations of total plasma *N-*glycome (TPNG) with type 2 diabetes have been reported, little is known on the role of TPNG in type 2 diabetes complications, a major cause of type 2 diabetes-related morbidity and mortality. Here, we assessed TPNG in relation to type 2 diabetes complications in subsamples of two Dutch cohorts using mass spectrometry (n=1815 in DiaGene and n=1518 in Hoorn Diabetes Care System).

**Research design and methods:**

Blood plasma samples and technical replicates were pipetted into 96-well plates in a randomized manner. Peptide:N-glycosidase F (PNGase F) was used to release *N-*glycans, whereafter sialic acids were derivatized for stabilization and linkage differentiation. After total area normalization, 68 individual glycan compositions were quantified in total and were used to calculate 45 derived traits which reflect structural features of glycosylation. Associations of glycan features with prevalent and incident microvascular or macrovascular complications were tested in logistic and Cox regression in both independent cohorts and the results were meta-analyzed.

**Results:**

Our results demonstrated similarities between incident and prevalent complications. The strongest association for prevalent cardiovascular disease was a high level of bisection on a group of diantennary glycans (A2FS0B; OR=1.38, p=1.34×10^−11^), while for prevalent nephropathy the increase in 2,6-sialylation on triantennary glycans was most pronounced (A3E; OR=1.28, p=9.70×10^−6^). Several other TPNG features, including fucosylation, galactosylation, and sialylation, firmly demonstrated associations with prevalent and incident complications of type 2 diabetes.

**Conclusions:**

These findings may provide a glance on how TPNG patterns change before complications emerge, paving the way for future studies on prediction biomarkers and potentially disease mechanisms.

**Significance of this study**

**What is already known about this subject?**

- Despite extensive preventive efforts, a substantial residual risk of macrovascular and microvascular complications in type 2 diabetes remains.
- Plasma *N*-glycans have been associated with type 2 diabetes itself and multiple risk factors for its complications (eg, lipids, smoking, inflammation).

**What are the new findings?**

- Fucosylation, galactosylation, sialylation, and bisection as main glycan features were associated with complications at baseline and follow-up.
- Of these, 13 and 15 features remained associated after false discovery rate correction for cardiovascular disease and nephropathy, respectively.
- Sialylation linkage type was associated with type 2 diabetes complications, showing positive associations for alpha2,6-linked and negative associations for alpha2,3-linked sialic acids.

**How might these results change the focus of research or clinical practice?**

- To improve diagnostics and treatment, we need more insights into pathophysiology. Our findings link typical total plasma *N-*glycome patterns to the development of complications, paving the way for future studies on prediction biomarkers and potential therapeutic approaches.

## Introduction

Type 2 diabetes is one of the most challenging health issues in the 21st century.^1^ The main burden of the disease results from long-term microvascular and macrovascular complications. These are important causes of disability, reduced quality of life, and death. Current treatment to prevent complications consists of strict glycemic control and preventing cardiovascular disease (CVD), by measures such as changes in lifestyle, blood pressure control, and lipid level optimization. Unfortunately, a substantial residual risk remains.^2^ The pathophysiology of type 2 diabetes and its complications is multifactorial and complex, involving genetic susceptibility, various metabolic pathways, lifestyle, and environmental factors.^1^ To design more effective prevention and treatment approaches, indepth pathophysiological insights are urgently needed. Also, in order to have a more rapid and accurate diagnosis, understanding of early biomarkers of diabetes complications is beneficial.

As opposed to the passive glycation of hemoglobin A1c (HbA1c) and the rather static, inherited nucleic acid sequence in our DNA, glycosylation of proteins results from active, enzymatically controlled processes. Glycans are present on most human proteins and are synthesized through a concerted action of several hundreds of enzymes. They have different functions in human biology and reflect genetic, metabolic, and environmental factors. Since they are abundantly present and can influence almost every biological process through subtle changes in their structure, glycosylation changes are thought to be involved in every major disease.^3 4^ Plasma *N*-glycans have been proposed as suitable candidates for disease biomarker research since they are very stable in healthy individuals over time,^5 6^ but do reflect physiological, pathological, or lifestyle changes in an individual.^3 4^

Plasma *N-*glycans have already been linked to metabolic syndrome, obesity, plasma lipid levels, blood pressure, smoking habits, inflammatory diseases, and type 2 diabetes itself.^7–12^

The association of the plasma *N-*glycome with complications, that is, retinopathy, nephropathy, and CVD, in type 2 diabetes has been sparsely investigated. Wittenbecher *et al*^13^ researched the ability of the plasma *N*-glycome to predict the incidence of type 2 diabetes and CVD and demonstrated that *N*-glycan predictors of cardiovascular endpoints only partially overlapped with those related to type 2 diabetes. Also, *N*-glycan profiles of patients with type 2 diabetes and subjects with metabolic syndrome showed changes in the presence of macrovascular complications.^14^ Here, we aimed to gain a deeper insight into the total plasma *N-*glycome (TPNG) in prevalent and incident retinopathy, nephropathy, and CVD in two large independent prospective cohorts of patients with type 2 diabetes, employing a novel, high-resolution analytical technique employing mass spectrometric detection of 68 different glycan structures.

## Research design and methods

Details on the cohorts, including definitions and biochemical measurements, have been described elsewhere.^15 16^

### Study populations

#### DiaGene study

The DiaGene study is a case–control study on type 2 diabetes from the region of Eindhoven, the Netherlands, and has previously been described in detail elsewhere.^15^ In total, 1886 cases and 854 controls were enrolled, with cases from both primary and secondary care. For the current study, only cases were included. Cases included in the current analyses have a mean follow-up of 7.0 years (95% CI 6.9 to 7.1, IQR 2.2).

#### Hoorn Diabetes Care System study

The Hoorn Diabetes Care System (Hoorn DCS) study has been described in detail elsewhere.^16^ In short, primary care patients with type 2 diabetes from the region of West Friesland in the Netherlands visited the DCS research center annually for routine diabetes care (n~14 000). Biobanking materials, anthropometric, clinical, biochemical, and data on annual examinations for microvascular and macrovascular complications have been collected for ~6000 persons who agreed to participate in the DCS biobanks.^16^ For this particular study, we randomly chose plasma samples of 1600 subjects who donated a sample in 2008/2009. The median follow-up was 8.5 years with a 95% CI of 2.1 to 9.2 and an IQR of 1.8.

The clinical characteristics of both cohorts are shown in table 1. Detailed characteristics of those with prevalent or incident complications are shown in online supplemental tables 1 and 2.

10.1136/bmjdrc-2021-002345.supp2 Supplementary data

**Table 1:** Hoorn DCS and DiaGene cohort characteristics

	DiaGene	Hoorn DCS
Number of participants	1815	1518
Female sex, % (n)	46.3 (841)	43.7 (664)
Age, years, mean (±SD)	65.2 (10.6)	64.5 (10.6)
Age of onset diabetes, years, mean (±SD)	54.9 (11.7)	57.3 (11.0)
BMI, kg/m^2^, mean (±SD)	29.5 (5.5)	30.4 (5.4)
HbA1c, % (SD)	7.0 (1.1)	6.8 (1.0)
HbA1c, mmol/mol, mean (±SD)	53.3 (11.6)	51 (11)
Systolic blood pressure, mm Hg, mean (±SD)	141.9 (18.8)	144 (19)
Diastolic blood pressure, mm Hg, mean (±SD)	77.4 (9.9)	76 (10)
Total cholesterol, mmol/L, mean (±SD)	4.3 (0.9)	4.6 (1.7)
Triglycerides, mmol/L, median (IQR)	1.4 (1.0–2.0)	1.6 (1.2–2.2)
HDL-c, mmol/L, mean (±SD)	1.2 (0.3)	1.2 (0.4)
LDL-c, mmol/L, mean (±SD)	2.5 (0.8)	2.6 (0.9)
Non-HDL-c, mmol/L, mean (±SD)	3.1 (0.9)	3.5 (1.5)
Smoking		
Never (n)	425	926
Former (n)	924	334
Current (n)	294	258
Complications		
Incident CVD, % (n case/n total)	10.1 (104/1034)	8.0 (97/1220)
Prevalent CVD, % (n case/n total)	34.6 (628/1669)	19.0 (288/1518)
Incident nephropathy, % (n case/n total)	20.1 (244/1214)	12.4 (158/1272)
Prevalent nephropathy, % (n case/n total)	20.2 (367/1621)	15.5 (236/1518)
Incident retinopathy, % (n case/n total)	16.1 (219/1361)	9.9 (126/1278)
Prevalent retinopathy, % (n case/n total)	16.0 (291/1714)	14.2 (214/1503)

Details on the cohorts, including definitions and biochemical measurements, have been described elsewhere.^15 16^

BMI, body mass index; CVD, cardiovascular disease; DCS, Diabetes Care System; HbA1c, hemoglobin A1c; HDL-c, high-density lipoprotein cholesterol; LDL-c, low-density lipoprotein cholesterol.

### Definitions

#### Type 2 diabetes

For both studies, the diagnosis of type 2 diabetes was defined in accordance with the American Diabetes Association (ADA) and WHO guidelines.^17 18^ Persons diagnosed with type 1 diabetes and other types of diabetes were excluded.

#### Retinopathy, nephropathy, and CVD

The definitions for microvascular and macrovascular events in the DiaGene and DCS study populations have been described previously.^15 16^ In short, for both studies, retinopathy was scored and graded according to the report of an ophthalmologist and fundus photography and for the current analyses scored as present or absent. Nephropathy was defined as microalbuminuria (albumin to creatinine ratio (ACR) ≥2.5 for men or ≥3.5 for women) present at two of three consecutive measurements, or when high microalbuminuria or macroalbuminuria was present at one measurement (ACR ≥12.5 for men or ≥17.5 for women). CVD was defined as myocardial infarction, percutaneous coronary intervention/coronary arterial bypass graft, cerebrovascular accident, transient ischemic attack, and peripheral arterial disease as derived from medical records and questionnaires.^15 16^

### *N*-glycome analysis and data quality control

The analysis method of the TPNG as applied on the DiaGene cohort has been previously described.^9 19^ Samples from the Hoorn DCS cohort were analyzed using a recently improved method,^20^ as described in detail elsewhere.^21^ In short, blood plasma samples were randomized over 96-well plates together with technical replicates, and glycans were released from proteins using the enzyme Peptide:N-glycosidase F (PNGase F). After glycan stabilization by sialic acid derivatization, released glycans were purified and spotted using an automated liquid handling platform. TPNG was measured by mass spectrometry employing matrix-assisted laser desorption/ionization with time-of-flight (DiaGene) or Fourier-transform ion cyclotron resonance (Hoorn DCS) analyzer. Mass spectra in both cohorts were checked for quality and excluded in case of low intensity. The intensity sum of 73 (DiaGene) or 68 (Hoorn DCS) directly measured glycan compositions was normalized to one. Seventy (DiaGene) and 68 (Hoorn DCS) direct glycan compositions were further used to calculate 45 derived traits based on their structural similarities (online supplemental table 3). Batch correction was then performed using the ComBat package in R. A total of 1815 DiaGene and 1518 DCS samples passed quality control and were used in the statistical analyses. By using pooled plasma replicates which were randomized over 96-well plates together with the clinical samples, method precision was assessed. Relative SDs of derived traits are reported in online supplemental table 3 and were on average 5.92% in DiaGene and 4.40% in DCS.

### Statistical analyses

Statistical analyses were done in R statistics V.3.6.0. Missing data on covariates (body mass index (BMI), high-density lipoprotein-cholesterol (HDL-c), non-HDL-c, systolic blood pressure, HbA1c, smoking, diabetes duration, and creatinine) were imputed using multivariate imputation by chained equations (R package MICE).^22^ When possible, clinical covariates from the year before and after the missing value were added to improve the imputation quality. The maximum count of imputations per variable was 172 in DiaGene and 20 in Hoorn DCS; all imputed variables had <7% missing values. The clinical characteristics after imputation are shown in online supplemental tables 4 and 5. The plasma *N-*glycans were Z-scaled before analysis to facilitate meta-analysis of both cohorts. Logistic regression models and Cox proportional hazards models served to analyze the prevalent and incident associations of the *N-*glycan traits (as independent variables) and retinopathy, nephropathy, and CVD as dependent variables, respectively. All analyses were corrected for age, sex, and their interaction (basic model or model 1). A full model was additionally corrected for BMI, HDL-c, non-HDL-c, systolic blood pressure, diabetes duration, HbA1c, and smoking for the nephropathy endpoint. In addition, correction for creatinine levels was included for retinopathy and CVD analyses. Non*-*HDL-c was calculated as total cholesterol minus HDL-c. Smoking comprised information on current, former, and never smoking. Heterogeneity between cohorts was also evaluated using I^2^ values as described previously.^23^ To summarize the results of the two independent cohorts, random-effects meta-analysis was performed on the entire set of 45 traits, including significant and non-significant associations using the R package meta.^24^ Correction for multiple testing with the Benjamini-Hochberg method^25^ at a false discovery rate (FDR) of 5% was applied. FDR-adjusted *p* values <0.05 were regarded as significant, unless explicitly stated as nominally significant *p* values.

## Results

### Cohort characteristics

In both cohorts combined, a total of 916 and 407 persons had prevalent and incident CVD, respectively. Six hundred and three persons had prevalent and 832 developed incident nephropathy, and 505 and 572 individuals with prevalent and incident retinopathy were included, respectively.

Details on the clinical characteristics of both studies are shown in table 1 and further details can be found in online supplemental tables 1 and 2. Correlations between *N*-glycan traits showed similar patterns for both cohorts (online supplemental figure 1). Bisection, except for bisecting *N-*acetylglucosamine within fucosylated non*-*sialylated diantennary glycans (A2FS0B), was mostly positively correlated with fucosylation and negatively correlated with galactosylation. Alpha2,6-sialylation of diantennary glycans (A2E) was negatively correlated with diantennary fucosylation (A2F, A2L0F), next to several positive correlations between structurally similar traits and negative correlations between complementary traits, such as α2,6-sialylation versus α2,3-sialylation. In the following, only meta-analyzed results are presented; however, the original outputs from the regression analyses per cohort can be provided by the corresponding author on request.

10.1136/bmjdrc-2021-002345.supp1 Supplementary data

### Plasma *N-*glycome associations with CVD

**Figure 1:**
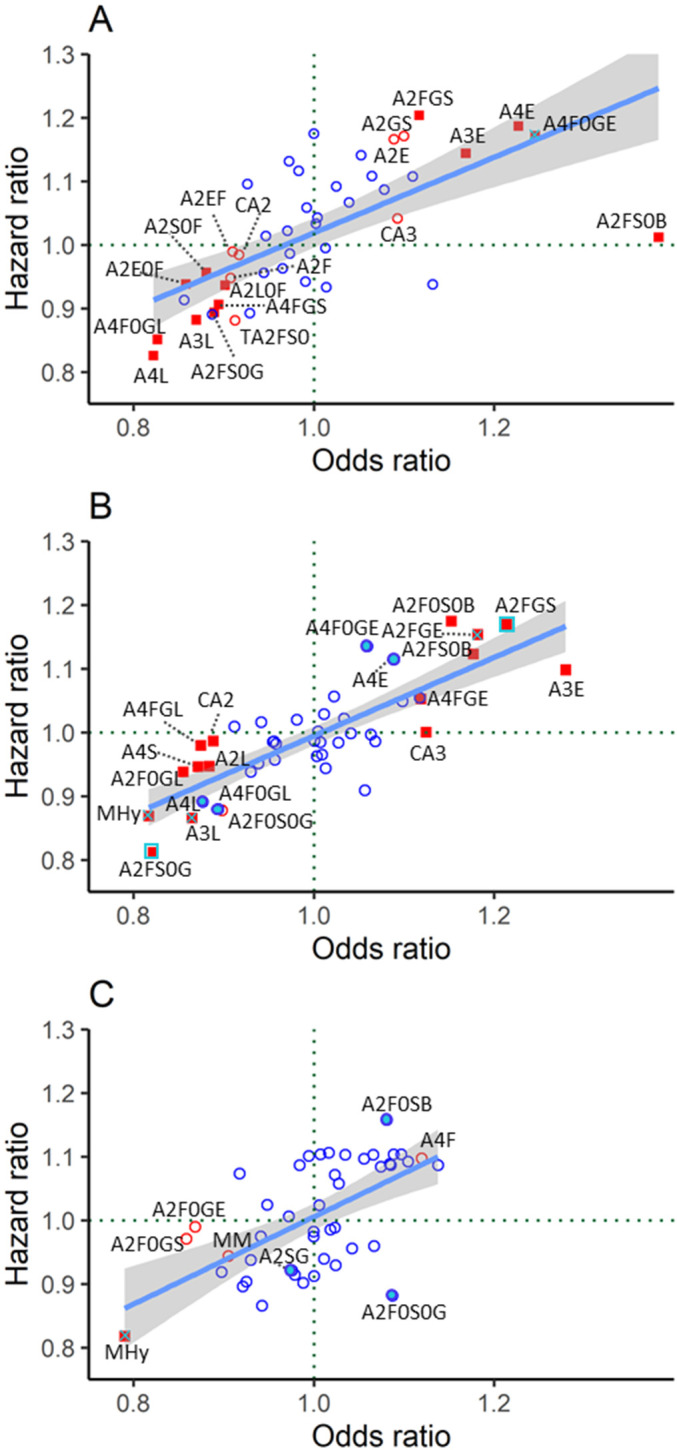
*N-*glycan-derived trait HR plotted versus OR for meta-analyzed data from DiaGene and Hoorn Diabetes Care System studies in the basic model (adjusted for age, sex, and age×sex interaction). (A) Cardiovascular disease, (B) nephropathy, and (C) retinopathy. Red-filled blue square: significant in prevalent and incident complications after FDR correction. Red-filled square with blue cross: significant in prevalent complications after FDR correction and in incident complications before FDR correction. Red-filled square: significant in prevalent complications after FDR correction. Blue-filled circle: significant in incident complications before FDR correction. Red unfilled circle: significant in prevalent complications before FDR correction. Blue unfilled circle: non-significant. Glycan derived trait abbreviations are described in table 2 and online supplemental table 3. FDR, false discovery rate.

At baseline, after age and sex correction (model 1), 13 *N*-glycans were associated with prevalent CVD (online supplemental table 6). One of the strongest associations found was for species with bisecting *N-*acetylglucosamine within fucosylated non*-*sialylated diantennary glycans (A2FS0B) (OR=1.38, p=1.34×10^−11^; online supplemental table 6, table 2 and figure 1A). Alpha2,3-linked sialylation, for example, A3L and A4F0GL, and diantennary fucosylated glycans (A2F and A2EF) were lower, while α2,6-linked sialylation in triantennary and tetra-antennary glycans (A3E and A4F0GE) was higher in patients with CVD. Moreover, A2FS0B, A4F0GE, A4E, A2E0F, A2S0F, A4F0GL, and A4L remained significant in the full model (online supplemental table 7 and online supplemental figure 2A).

**Table 2:** Meta-analyzed associations of selected plasma *N*-glycans with type 2 diabetes complications and with type 2 diabetes

Derived traits	Description†	Cardiovascular disease				Nephropathy				Retinopathy				Type 2 diabetes vs healthy controls*	
		Prevalent (logistic)		Incident (cox)		Prevalent (logistic)		Incident (cox)		Prevalent (logistic)		Incident (cox)		(logistic)	
		OR	*p* value	HR	*p* value	OR	*p* value	HR	*p* value	OR	*p* value	HR	*p* value	OR	*p* value
Complexity															
MHy	Ratio of high-mannose to hybrid glycans.	1.01	0.903	0.99	0.947	0.82	**1.20×10^−3^**	0.87	0.089	0.79	**0.047**	0.82	0.292	0.86	0.213
CA2	Relative abundance of A2 glycans within complex-type glycans.	0.92	0.104	0.98	0.947	0.89	**0.046**	0.99	0.962	1.02	0.921	1.07	0.385	0.85	**6.09×10^−3^**
CA3	Relative abundance of A3 glycans within complex-type glycans.	1.09	0.104	1.04	0.808	1.12	**0.046**	1.00	0.998	0.98	0.922	0.92	0.370	1.21	**1.39×10^−3^**
Fucosylation (F)															
A2E0F	In A2 glycans without α2,6-linked sialic acid.	0.86	**2.23×10^−3^**	0.94	0.718	0.95	0.527	0.99	0.962	0.95	0.543	1.02	0.854	0.76	**3.73×10^−6^**
A2L0F	In A2 glycans without α2,3-linked sialic acid.	0.90	**0.049**	0.94	0.718	0.96	0.821	0.99	0.962	1.06	0.543	1.10	0.385	0.79	**1.94×10^−5^**
A2S0F	In non-sialylated A2.	0.88	**0.013**	0.96	0.752	0.94	0.383	1.02	0.962	0.97	0.902	1.01	0.944	0.86	**0.010**
Bisection (B)															
A2FS0B	In fucosylated non-sialylated A2.	1.38	**1.34×10^−11^**	1.01	0.947	1.18	**5.35×10^−3^**	1.12	0.229	1.03	0.921	1.06	0.440	1.54	**1.98×10^−12^**
A2F0S0B	In non-fucosylated non-sialylated A2.	1.13	0.180	0.94	0.718	1.15	**0.014**	1.17	0.282	0.99	0.974	1.10	0.337	1.16	**0.011**
Galactosylation (G)															
A2FS0G	In fucosylated non-sialylated A2.	0.89	**0.049**	0.89	0.626	0.82	**2.09×10^−3^**	0.81	**0.0110**	0.94	0.543	0.87	0.385	0.86	**0.014**
Sialylation (S)															
A4FGS	Per galactose in fucosylated A4.	0.89	**0.040**	0.91	0.626	0.91	0.320	1.01	0.962	1.00	1.00	0.97	0.854	0.80	**1.03×10^−4^**
A2FGS	Per galactose in fucosylated A2.	1.12	**0.041**	1.20	0.626	1.21	**1.20×10^−3^**	1.17	**0.047**	1.09	0.456	1.09	0.385	1.77	**2.65×10^−8^**
A4S	In A4.	0.93	0.342	0.89	0.626	0.87	**0.015**	0.95	0.886	1.02	0.922	0.99	0.854	0.74	**2.08×10^−7^**
α2,3-sialylation per antenna (L)															
A4F0GL	Per galactose in non-fucosylated A4.	0.83	**2.23×10^−3^**	0.85	0.368	0.89	0.231	0.88	**0.089**	0.98	0.921	0.91	0.337	0.68	**2.06×10^−10^**
A4L	In A4.	0.82	**0.013**	0.83	0.353	0.88	0.217	0.89	0.146	1.00	1.00	0.91	0.337	0.64	**3.50×10^−13^**
A3L	In A3.	0.87	**0.034**	0.88	0.626	0.86	**0.010**	0.87	0.146	0.99	0.953	0.90	0.337	0.61	**5.73×10^−14^**
A2F0GL	Per galactose in non-fucosylated A2.	0.94	0.551	0.96	0.752	0.86	**8.44×10^−3^**	0.94	**6.48×10^−1^**	1.04	0.691	0.96	0.553	0.81	**1.18×10^−4^**
A4FGL	Per galactose in fucosylated A4.	0.89	0.370	0.89	0.626	0.87	**0.015**	0.98	0.962	1.02	0.921	0.93	0.385	0.67	**2.11×10^−3^**
A2L	In A2.	1.00	0.974	1.03	0.808	0.88	**0.039**	0.95	0.680	1.07	0.479	0.96	0.594	0.89	0.102
α2,6-sialylation per antenna (E)															
A4F0GE	Per galactose in non-fucosylated A4.	1.25	**4.88×10^−6^**	1.17	0.368	1.06	0.797	1.14	**0.089**	1.03	0.811	1.10	0.337	1.31	**3.73×10^−6^**
A4E	In A4.	1.23	**8.65×10^−4^**	1.19	0.626	1.09	0.527	1.12	0.154	1.02	0.922	1.11	0.337	1.42	**8.76×10^−9^**
A3E	In A3	1.17	**2.23×10^−3^**	1.14	0.626	1.28	**9.70×10^−6^**	1.10	0.394	0.92	0.456	1.07	0.385	1.54	**1.71×10^−11^**
A2FGE	Per galactose in fucosylated A2.	1.05	0.370	1.14	0.718	1.18	**3.77×10^−3^**	1.15	0.059	1.08	0.456	1.09	0.545	1.72	**1.98×10^−3^**
A4FGE	Per galactose in fucosylated A4.	1.08	0.682	1.09	0.718	1.12	**0.046**	1.05	0.851	0.98	0.953	1.09	0.337	1.52	0.094

Selection of glycan traits is based on derived traits that were FDR-significant (p<0.05) at least in one of the complications (basic model: age, sex, age×sex interaction).

Significant associations are marked bold.

*The two utmost right columns show the results of meta-analyzed associations from a logistic regression analysis of the same glycan traits measured in patients with type 2 diabetes versus healthy controls from the DiaGene and Hoorn Diabetes Care System cohorts, as previously reported by Singh *et al.*^21^

†Glycan trait description: see abbreviation footnotes.

A2, diantennary; A3, triantennary; C, within complex; FDR, false discovery rate; T, within total.

In Cox regression analysis for incident CVD, none of the nominally significant associations remained significant after FDR correction (online supplemental tables 6 and 7). When comparing the associations of prevalent and incident CVD in model 1, the three nominally significant traits at follow-up, that is, A4F0GE, A4F0GL, and A4L, showed similar trends and effect sizes to the associations found at baseline (figure 1A and table 2).

For comparison, the last two columns in table 2 show the associations of *N*-glycan traits with type 2 diabetes derived from Singh *et al*.^21^

### Plasma *N-*glycome associations with nephropathy

In logistic regression model 1, 15 derived traits were associated with prevalent nephropathy, overall showing increased α2,6-sialylation, bisection and glycan branching, and decreased α2,3-sialylation and IgG-related galactosylation (table 2 and online supplemental table 8). The strongest association was observed for α2,6-sialylation of triantennary glycans (A3E) (OR=1.28, p=9.70×10^−6^). Adding covariates in the full model did affect *p* values and effect sizes when compared with model 1, but did not alter the direction of the associations (online supplemental table 9 and online supplemental figure 2B).

In Cox regression model 1, the derived traits A2FS0G and A2FGS were associated with incident nephropathy (table 2). In the full model, A2FS0G and A2FGS were only nominally significant, in addition to the ratio of high-mannose to hybrid glycans (MHy), A3S, and A2F0GS (online supplemental tables 8 and 9). Overall, glycan trait associations showed similar trends and correlated between prevalent and incident nephropathy in model 1 (figure 1B and table 2).

### Plasma *N-*glycome associations with retinopathy

In the basic model, MHy was associated with prevalent retinopathy (OR=0.79, p=4.68×10^−2^) (table 2 and figure 1C). In the full model, no associations with prevalent retinopathy were found (online supplemental figure 2C).

In Cox regression analysis for incident retinopathy, only nominally significant associations were found in both models (online supplemental tables 10 and 11). Overall, MHy showed similar effect sizes for prevalent and incident retinopathy in model 1 (figure 1C and table 2).

## Discussion

In our analyses on the associations of TPNG with prevalent and incident type 2 diabetes complications in two large independent cohorts, we found multiple associated traits, with similar patterns in both cohorts. The strongest associations were demonstrated for CVD and nephropathy, with increased bisection of IgG-related glycans, increased α2,6-sialylated species, and decreased galactosylation of IgG-related glycans. Additionally, a decrease in fucosylated and in the ratio of high-mannose to hybrid glycans was observed only for CVD and nephropathy, respectively.

Some of these associations (higher α2,6-sialylation and bisection, lower α2,3-sialylation and fucosylation) showed overlaps with findings in diabetes mellitus versus controls (table 2), which may be a feature of type 2 diabetes or may have been the driving force of complications within diabetes.

### Cardiovascular disease

One of the strengths of our study is the ability to distinguish sialic acid linkage isomers. For both prevalent and incident CVD we found a positive association of diantennary, triantennary, and tetra-antennary α2,6-sialylation (ie, A4F0GE, A4E, A3E, and A2E) and a negative association of triantennary and tetra-antennary α2,3-sialylation (ie, A4F0GL, A4L, and A3L). A similar pattern has also been reported for type 2 diabetes,^9 21^ in contrary to other inflammatory conditions, such as inflammatory bowel disease or colorectal cancer, where both α2,6-sialylation and α2,3-sialylation increased.^26 27^ Therefore, while higher α2,6-sialylation might be reflecting systemic inflammation which is inherent to all the aforementioned conditions, α2,3-sialylation may reflect disease-specific changes. More specifically, in diabetes, hyperglycemia leads to increased production of liver acute phase proteins, which carry both α2,6-linked and α2,3-linked sialic acids due to increased sialyltransferase activity.^28 29^ Moreover, CVD is accompanied by an elevation of sialylated acute phase proteins such as fibrinogen and ceruloplasmin.^30^ Plasma levels of alpha-2-Heremans-Schmid (HS)-glycoprotein were associated with obesity and type 2 diabetes,^31 32^ and it appears to promote calcification in coronary artery disease.^33^ In terms of the specific sialic acid linkage isomers, we can speculate on the role of beta-galactoside alpha-2,6-sialyltransferase-1 enzyme which is encoded by the ST6GAL1 gene, a type 2 diabetes genome-wide association study (GWAS)-confirmed risk gene.^34^ Higher activity of this enzyme in CVD in the context of type 2 diabetes could potentially lead to a positive association with α2,6-sialylation. Overall, our findings of increasing α2,6-sialylation and decreasing α2,3-sialylation in prevalent and incident CVD patients with type 2 diabetes point to a possible role of these signatures in the etiology of CVD.

Also, we found a decrease in fucosylated, non*-*sialylated diantennary (A2S0F) and galactosylation of fucosylated, non*-*sialylated diantennary (A2FS0G) structures, and a strong increase in bisected fucosylated non*-*sialylated diantennary (A2FS0B) structures associated with both prevalent and incident CVD. These traits are mostly derived from IgG.^29^ The absence of fucose enhances the antibody-dependent cytotoxicity of IgG in vitro.^4 35^ To a lesser extent, higher levels of bisecting *N-*acetylglucosamine on IgG are often associated with a greater affinity for Fcγ-receptors, and consequently with enhanced antibody-dependent cell-mediated cytotoxicity.^36^ Similar to our data, previous work in the DiaGene cohort demonstrated that bisected glycans on IgG were increased in type 2 diabetes,^37^ which shows partially opposing direction for addition of bisection and fucosylation. Decreased IgG galatosylation (A2FS0G) in our study agrees with previous reports in inflammatory diseases.^4^ Taken together, our findings on IgG-related glycosylation changes and overall α2,6-sialylation increase seem to reflect inflammation.

### Nephropathy

We observed an increase in relative abundance of triantennary glycans within complex-type glycans (CA3) and a decrease in the relative abundance of diantennary glycans (CA2) in prevalent nephropathy, while the MHy was decreased in both incident and prevalent nephropathy in the basic model. Similar findings have previously been reported in a glycomics study on hyperglycemia and kidney function in type 1 diabetes.^38^ The positive association of CA3 might be due to an increased production of uridine diphosphate *N*-acetylglucosamine in hyperglycemia, which in turn increases the production of complex *N-*glycans. Increased *N*-glycan branching, corresponding to higher CA3, has also been shown to regulate the epidermal growth factor receptor and transforming growth factor-β pathways that are implicated in diabetic kidney disease.^39 40^ However, in our full model the associations of CA2 and CA3 were no longer significant.

High-mannose-type glycans are for a large part derived from apolipoprotein B100 and IgM.^29^ Increased apolipoprotein B100 was previously found to be independently associated with progression of chronic kidney disease in patients with diabetes.^41^ However, patients in our study were on lipid-lowering therapy. Since patients with or at risk of nephropathy are at high risk of macrovascular complications, more stringent lipid-lowering therapy measures in these patients might very well contribute to the inverse association of the MHy trait with nephropathy in our study. Of note, after correcting for non-HDL-c, the association remained significant.

We also found positive and negative associations in diantennary and tetra-antennary sialylated structures, respectively. Barrios *et al*^42^ showed that the major sialylated (diantennary) glycan and the percentage of sialylated IgG without bisecting *N-*acetylglucosamine decreased in individuals with chronic kidney disease. Their analytical method, however, did not allow them to differentiate and quantify the types of sialic acid linkages. Our analytical technique revealed that several α2,3-sialylation (L) traits had a negative association, whereas α2,6-sialylated (E) glycans were positively associated with nephropathy in both prevalent and incident cases. Similar associations have been reported for type 2 diabetes glycome^9^ and can be partly linked to inflammation, for example, A3E, or potentially disease-specific glycosylation changes, in case of α2,3-sialylation.^10^

Finally, bisected glycans without (A2F0S0B) and with (A2FS0B) fucosylation cross-sectionally showed a positive association with nephropathy in our study. The latter is similar to a finding in IgG glycosylation that showed increased bisection in patients with chronic kidney disease.^42^

In contrast, galactosylated, non-fucosylated, non-sialylated diantennary (A2F0S0G) and IgG-related galactosylated (A2FS0G) species showed a strong negative association with nephropathy both cross-sectionally and prospectively. A previous TPNG study by Adua *et al*^43^ showed lower galactosylated fucosylated and higher galactosylated sialylated glycans in patients with type 2 diabetes and chronic kidney disease (CKD) than those without CKD. Our findings are moreover in line with a study showing decreased IgG galactosylation to be associated with complement activation and renal damage.^44^

### Retinopathy

For retinopathy, the strongest association and the only FDR-corrected significant association at baseline and follow-up were with the ratio of high-mannose to hybrid glycans in model 1, with the same but non-significant trend visible in the full model. As discussed for nephropathy, this negative association of MHy might be due to a decrease of high-mannose rich apolipoprotein B100.

The strengths of our study are that it is the first to investigate the association of TPNG with multiple macrovascular and microvascular complications in type 2 diabetes, in two large independent cohorts with follow-up. We used an automated method with high-resolution mass spectrometry techniques. Moreover, sialic acid derivatization enabled us to quantify and distinguish sialic acid linkage-specific changes, which is a unique feature not investigated in other studies.

A few limitations are worth mentioning. The effect of medication use as per complication could not be investigated here due to statistical power limitations. Recently we demonstrated associations of TPNG and medication use,^21^ which may play a role in the current findings. However, medication use is also strongly related to the presence or absence of diabetes complications, which makes it difficult to correct for medication use without correcting for the outcome. Also, the number of incident cases for each complication was limited in CVD and retinopathy, constraining the power of these analyses. Thus, we cannot exclude that potential associations remained undetected. Future studies with even larger sample sizes will enable more detailed studies on associations and interactions including medication use. Despite the ability to separate sialic acid linkage isomers, our technique does not distinguish between other isomeric structures, such as galactose linkage isomers.

To conclude, we found multiple associations of TPNG with CVD and nephropathy in type 2 diabetes at baseline and at follow-up. Those glycan traits belong to the main glycosylation features: complexity, fucosylation, bisection, galactosylation, and linkage-specific sialylation. These results can be used for future translational research and provide novel insights into type 2 diabetes complications. Moreover, this would help to gain additional understanding on the potential of TPNG patterns as biomarkers for type 2 diabetes complications.

## Data Availability

Data are available upon reasonable request. The data that support the findings of this study are available on request from the corresponding author, who takes responsibility for the integrity of the data and the accuracy of the data analysis. The data are not publicly available due to them containing information that could compromise research participant privacy/consent.
