# Supplementary material for: Plasma protein N-glycosylation is associated with cardiovascular disease, nephropathy, and retinopathy in type 2 diabetes
Source: BMJ Open Diabetes Res Care. 2021 Oct 13;9(1):e002345. doi: 10.1136/bmjdrc-2021-002345 (PMC8515459; doi:10.1136/bmjdrc-2021-002345)
Supplement: Supplementary data [file bmjdrc-2021-002345supp002.pdf]

The following material is supplementary to the manuscript:

Plasma protein *N*-glycosylation is associated with cardiovascular disease, nephropathy, and retinopathy in type 2 diabetes.

Elham Memarian [a,b *], Leen M 't Hart [c,d,e *], Roderick C. Slieker [c,d], Roosmarijn F.L. Lemmers [f], Amber A van der Heijden [g], Femke Rutters [h], Giel Nijpels [g], Emma Schoep [c], Aloysius G. Lieverse [i], Eric J.G. Sijbrands [f], Manfred Wuhrer [a], Mandy van Hoek [f **], Viktoria Dotz [a **,#]

[a] Center for Proteomics and Metabolomics, Leiden University Medical Center, the Netherlands

[b] Genos Glycoscience Research Laboratory, Zagreb, Croatia

[c] Department of Cell and Chemical Biology, Leiden University Medical Center, the Netherlands

[d] Department of Epidemiology and Biostatistics, Amsterdam University Medical Center, location VUMC, Amsterdam, The Netherlands

[e] Department of Biomedical Data Sciences, Section Molecular Epidemiology, Leiden University Medical Center, the Netherlands

[f] Department of Internal Medicine, Erasmus MC, University Medical Center, Rotterdam, the Netherlands

[g] Amsterdam UMC, Vrije Universiteit Amsterdam, Department of General Practice Medicine, Amsterdam Public Health Research Institute, Amsterdam, the Netherlands

[h] Amsterdam UMC, Vrije Universiteit Amsterdam, Department of Epidemiology and Data Science, Amsterdam Public Health Research Institute, Amsterdam, the Netherlands

[i] Department of Internal Medicine, Maxima Medical Center, Eindhoven, the Netherlands

*These authors contributed equally
**These authors contributed equally

# Current address: BioTherapeutics Analytical Development, Janssen Biologics BV, Einsteinweg 101, 2333 CB Leiden, The Netherlands

Corresponding author:
Mandy van Hoek, ORCIDID: 0000-0002-2957-5436, Department of Internal Medicine, Erasmus MC – University Medical Center Rotterdam, Doctor Molewaterplein 40, 3015 GD, Rotterdam, The Netherlands, m.vanhoek@erasmusmc.nl, Tel.: +31 633343603

Supplementary Table 1. DiaGene cohort characteristics. Details on the cohorts including definitions and biochemical measurements have been described elsewhere. [15,16]

	**DiaGene Study-All**	**DiaGene Study prevalent CVD**	**DiaGene Study incident CVD**	**DiaGene Study prevalent Nephropathy**	**DiaGene Study incident Nephropathy**	**DiaGene Study prevalent Retinopathy**	**DiaGene Study incident Retinopathy**
Number of participants	1815	628	104	367	244	291	219
Age, yr, mean (±sd)	65.2 (10.6)	68.6 (9.2)	65.2 (9.4)	65.8 (10.14)	68.56 (9.58)	67.61 (9.68)	64.28 (10.80)
Female sex, %(n)	46.3 (841)	36.8 (231)	43 (45)	31.1 (114)	42.6 (104)	38.5 (112)	45.7 (100)
Age of onset diabetes, yr, mean (±sd)	54.9 (11.7)	57.2 (11.3)	52.8 (9.9)	54.0 (11.4)	58.24 (10.99)	50.2 (10.3)	51.00 (11.79)
Duration of diabetes, yr, median (IQR)	7.99 (2.47-14.13)	9.53 (4.02-16.65)	10.05 (5.46-16.72)	11.70 (6.61-18.27)	8.46 (4.12-15.43)	16.71 (10.87-23.05)	11.61 (7.09-17.26)
BMI, kg/m2, mean (±sd)	29.5 (5.5)	30.36 (4.90)	29.88 (4.82)	31.38 (5.78)	30.49 (5.59)	31.04 (5.79)	31.37 (5.63)
Total Cholesterol, mmol/L, mean (±sd)	4.29 (0.93)	4.15 (0.86)	4.47 (0.91)	4.14 (0.91)	4.24 (0.99)	4.16 (0.87)	4.11 (0.86)
Triglycerides, mmol/L, median (IQR)	1.42 (1.02-2.03)	1.45 (1.02-2.07)	1.40 (1.02-2.20)	1.54 (1.10-2.23)	1.52 (1.10-2.27)	1.39 (0.97-1.87)	1.44 (1.01-2.03)
HDL-c, mmol/L, mean (±sd)	1.17 (0.32)	1.12 (0.31)	1.23 (0.34)	1.12 (0.31)	1.14 (0.29)	1.20 (0.35)	1.17 (0.35)
LDL-c, mmol/L, mean (±sd)	2.45 (0.82)	2.34 (0.74)	2.56 (0.90)	2.24 (0.76)	2.40 (0.89)	2.26 (0.74)	2.25 (0.74)
Non-HDL-c, mmol/L, mean (±sd)	3.12 (0.90)	3.01 (0.84)	3.25 (0.94)	3.02 (0.88)	3.10 (0.99)	2.95 (0.84)	2.93 (0.82)
Creatinine, µmol/L, mean (±sd)	83.44 (28.65)	90.83 (33.78)	80.79 (30.23)	98.06 (40.80)	87.52 (29.88)	91.30 (32.68)	81.00 (26.37)
HbA1c, % (sd)	7.03 (1.06)	7.02 (1.00)	7.30 (1.15)	7.26 (1.17)	7.06 (1.09)	7.54 (1.09)	7.53 (1.31)
HbA1c, mmol/mol, mean (±sd)	53.31 (11.58)	53.24 (10.88)	56.33 (12.56)	55.89 (12.75)	53.70 (11.94)	58.91 (11.89)	58.80 (14.27)
Systolic blood pressure, mmHg, mean (±sd)	141.93 (18.79)	142.36 (19.72)	139.88 (16.58)	142.63 (19.73)	144.53 (18.19)	139.41 (19.31)	140.95 (17.38)
Diastolic blood pressure, mmHg, mean (±sd)	77.38 (9.87)	75.73 (10.18)	76.76 (9.58)	76.03 (9.79)	77.56 (9.43)	74.89 (9.18)	77.44 (10.34)
Smoking, no/former/yes, %	23.4/ 50.9/ 16.2	17.7/ 59.6/ 16.4	24.0/ 50.0/ 19.2	17.2/ 57.2/ 16.9	17.6/ 55.7/ 16.4	26.5/ 49.1/ 13.4	25.1/ 48.9/ 16.0
Insulin use, %(n)	29.8 (541)	33 (207)	34.6 (36)	39.5 (145)	27.9 (68)	55.7 (162)	50.7 (111)
Oral glucose-lowering medication, %(n)	61.1 (1109)	61.1 (384)	53.8 (56)	63.5 (233)	66.0 (161)	48.5 (141)	62.6 (137)
High-ceiling diuretics, %(n)	6.9 (125)	11.6 (73)	6.7 (7)	11.2 (41)	8.6 (21)	10.3 (30)	5.5 (12)
Lipid modifying agents, %(n)	65.8 (1194)	75.0 (471)	67.3 (70)	67.6 (248)	73.8 (180)	62.9 (183)	69.9 (153)
Renin-angiotensin system (RAS)-inhibitor, %(n)	53.3 (968)	65.4 (411)	51.0 (53)	71.7 (263)	61.9 (151)	60.1 (175)	54.8 (120)
Other antihypertensive medication, %(n)	13.3 (242)	14.5 (91)	11.5 (12)	8.7 (32)	13.1 (32)	8.6 (25)	12.3 (27)
Anticoagulant medication, %(n)	40 (726)	73.4 (461)	28.8 (30)	55 (202)	48.8 (119)	48.8 (142)	44.3 (97)
Biguanides, %(n)	51.1 (927)	51.1 (321)	43.3 (45)	54 (198)	57.0 (139)	42.6 (124)	56.6 (124)
incident CVD, % (n-case/n-total)	10.1 (104/1034)	31.1 (195)		25.1 (92)		29.6 (86)	
prevalent CVD, % (n-case/n-total)	34.6 (628/1669)	100 (628)		46.3 (170)		41.9 (122)	
incident Nephropathy, % (n-case/n-total)	20.1 (244/1214)	47.0 (295)		94.3 (346)		57.7 (168)	
prevalent Nephropathy, % (n-case/n-total)	20.2 (367/1621)	27.1 (170)		100 (367)		44.3 (129)	
incident Retinopathy, % (n-case/n-total)	16.1 (219/1361)	26.9 (169)		39.0 (143)		75.6 (220)	
prevalent Retinopathy, % (n-case/n-total)	16.0 (291/1714)	19.4 (122)		35.1 (129)		100 (291)	

Supplementary Table 2. Hoorn DCS cohort characteristics. Details on the cohorts including definitions and biochemical measurements have been described elsewhere. [15,16]

	Hoorn DCS study-All	Hoorn DCS study prevalent CVD	Hoorn DCS study incident CVD	Hoorn DCS study prevalent Nephropathy	Hoorn DCS study incident Nephropathy	Hoorn DCS study prevalent Retinopathy	Hoorn DCS study incident Retinopathy
Number of participants	1518	288	97	236	158	214	126
Age, yr, mean (±sd)	64.5 (10.6)	69.0 (9.0)	67.1 (9.2)	66.0 (11.1)	66.2 (10.5)	66.5 (10.8)	64.4 (10.9)
Female sex, %(n)	43.74 (664)	28.12 (81)	43.30 (42)	27.97 (66)	31.65 (50)	41.12 (88)	43.65 (55)
Age of onset diabetes, yr, mean (±sd)	57.3 (11.0)	61.0 (9.9)	59.9 (10.9)	56.6 (11.6)	58.8 (11.6)	55.6 (11.9)	55.8 (10.5)
Duration of diabetes, yr, median (IQR)	6.0 (2.9-9.9)	7.0 (3.2-10.9)	5.6 (2.3-10.5)	8.3 (5.0-12.4)	6.0 (2.6-10.0)	9.8 (6.4-14.3)	6.9 (3.0-12.0)
BMI, kg/m2, mean (±sd)	30.4 (5.4)	29.9 (5.1)	29.9 (4.7)	31.1 (6.1)	30.5 (5.3)	30.2 (5.7)	30.9 (5.3)
Total Cholesterol, mmol/L, mean (±sd)	4.63 (1.65)	4.35 (0.89)	4.78 (0.88)	4.53 (0.99)	4.48 (1.01)	4.61 (1.04)	4.59 (0.99)
Triglycerides, mmol/L, median (IQR)	1.58 (1.15-2.19)	1.63 (1.20-2.22)	1.56 (1.13-2.24)	1.75 (1.20-2.37)	1.70 (1.27-2.18)	1.59 (1.15-2.13)	1.69 (1.25-2.56)
HDL-c, mmol/L, mean (±sd)	1.18 (0.39)	1.13 (0.31)	1.21 (0.37)	1.10 (0.29)	1.12 (0.31)	1.17 (0.33)	1.14 (0.33)
LDL-c, mmol/L, mean (±sd)	2.63 (0.89)	2.41 (0.75)	2.75 (0.75)	2.59 (0.83)	2.51 (0.87)	2.64 (0.89)	2.54 (0.79)
Non-HDL-c, mmol/L, mean (±sd)	3.46 (1.48)	3.22 (0.85)	3.58 (0.87)	3.43 (1.01)	3.36 (0.99)	3.44 (1.03)	3.45 (0.95)
Creatinine, µmol/L, mean (±sd)	79.78 (22.24)	87.30 (23.94)	81.23 (18.27)	87.05 (33.29)	85.91 (22.15)	80.71 (19.29)	80.99 (33.95)
HbA1c, % (±sd)	6.8 (1.0)	6.9 (1.1)	6.8 (1.1)	7.1 (1.2)	6.9 (1.1)	7.1 (1.2)	7.4 (1.4)
HbA1c, mmol/mol, mean (±sd)	51 (11)	52 (12)	51 (12)	54 (13)	52 (12)	54 (12)	57 (15)
Systolic blood pressure, mmHg, mean (±sd)	144 (19)	145(20)	150 (20)	148 (21)	148 (20)	148(19)	148 (20)
Diastolic blood pressure, mmHg, mean (±sd)	76 (10)	74 (10)	77 (10)	77 (10)	77 (10)	77 (10)	77 (9)
Smoking, no/former/yes, %	61/ 22/ 17	57/ 28/ 15	54/ 26/ 21	52/ 21/ 27	58/ 29/ 13	65/ 19/ 16	62/ 24/ 14
Insulin use, %(n)	25.2 (382)	29.2 (84)	22.7 (22)	36.4 (86)	27.2 (43)	40.2 (86)	37.3 (47)
Oral glucose-lowering medication, %(n)	73.1 (1110)	72.6 (209)	76.3 (74)	75.8 (179)	81.0 (128)	73.4 (157)	82.5 (104)
High-ceiling diuretics, %(n)	7.9 (120)	13.5 (39)	7.2 (7)	15.7 (37)	5.7 (9)	11.2 (24)	7.1 (9)
Lipid modifying agents, %(n)	70.2 (1066)	87.8 (253)	62.9 (61)	72.9 (172)	75.9 (120)	73.4 (157)	65.1 (82)
Renin-angiotensin system (RAS)-inhibitor, %(n)	39.5 (599)	50.7 (146)	43.3 (42)	52.5 (124)	37.3 (59)	43.5 (93)	38.1 (48)
Other antihypertensive medication, %(n)	21.3 (323)	33.3 (96)	24.7 (24)	35.2 (83)	25.3 (40)	25.2 (54)	20.6 (26)
Anticoagulant medication, %(n)	3.1 (47)	9.4 (27)	1.0 (1)	4.2 (10)	1.9 (3)	3.7 (8)	2.4 (3)
Biguanides, %(n)	67.5 (1024)	68.1 (196)	68.0 (66)	69.9 (165)	76.6 (121)	65.9 (141)	79.4 (100)
incident CVD, % (n-case/n-total)	8.0 (97/1220)						
prevalent CVD, % (n-case/n-total)	19.0 (288/1518)						
incident Nephropathy, % (n-case/n-total)	12.4 (158/1272)						
prevalent Nephropathy, % (n-case/n-total)	15.5 (236/1518)						
incident Retinopathy, % (n-case/n-total)	9.9 (126/1278)						
prevalent Retinopathy, % (n-case/n-total)	14.2 (214/1503)						

Supplementary Table 3. Derived trait description, depiction, and calculation. Within the depictions, divisions have been indicated by braces, exclusions by a cross, whereas numerical values indicate compositional limitations (*i.e.* 5-9 as the possible number of mannoses, 1-4 N-acetylhexosamines, or 2 fucoses). Trait abbreviations: M = mannose; Hy = hybrid species; T = within total; C = within complex species; F = deoxyhexose (fucose); G = galactose; S = N-acetylneuraminic acid (sialic acid); E = α2,6-linked sialic acid; L = α2,3-linked sialic acid; H = hexose (mannose or galactose) ; N = N-acetylhexosamine (N-acetylglucosamine: GlcNAc).

Derived trait	Description	Depiction	Calculation	Relative intensity VisuCon standard plasma_DiaGene (N=149)			Relative intensity VisuCon standard plasma_Hoorn DCS (N=78)		
**Complexity**				**mean**	**SD**	**RSD**	**mean**	**SD**	**RSD**
**MM**	Average number of mannoses on high mannose type glycans	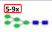	MM = (5 * (H5N2) + 6 * (H6N2) + 7 * (H7N2) + 8 * (H8N2) + 9 * (H9N2) + 10 * (0)) / (H5N2 + H6N2 + H7N2 + H8N2 + H9N2)	7.8250	0.12727234	1.63%	7.587083	0.08479	1.12%
**MHy**	The ratio of high-mannose to hybrid glycans	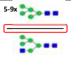	MHy = (H5N2 + H6N2 + H7N2 + H8N2 + H9N2) / (H5N3 + H6N3 + H5N3E1 + H6N3E1)	3.3010	0.8767	26.56%	2.389037	0.334529	14.00%
**CA2**	Relative abundance of diantennary glycans within complex type glycans		CA2 = (H3N4F1 + H4N4 + H3N5 + H4N4F1 + H5N4 + H3N5F1 + H4N5 + H5N4F1 + H4N4E1 + H4N5F1 + H5N5 + H5N4L1 + H4N4F1E1 + H5N4E1 + H5N5F1 + H4N5E1 + H5N4F1L1 + H5N4F1E1 + H4N5F1E1 + H5N5E1 + H5N4L2 + H5N4E1L1 + H5N4E2 + H5N5F1E1 + H5N4F1L2 + H5N4F1E1L1 + H5N4F1E2 + H5N5E2 + H5N5F1E1L1 + H5N5F1E2) / (H3N3E1 + H3N4F1 + H4N4 + H3N5 + H3N3F1E1 + H4N3E1 + H4N4F1 + H5N4 + H3N5F1 + H4N5 + H5N4F1 + H4N4E1 + H4N5F1 + H5N5 + H5N4L1 + H4N4F1E1 + H5N4E1 + H5N5F1 + H4N5E1 + H5N4F1L1 + H5N4F1E1 + H4N5F1E1 + H5N5E1 + H5N4L2 + H5N4E1L1 + H5N4E2 + H5N5F1E1 + H6N5E1 + H5N4F1L2 + H5N4F1E1L1 + H5N4F1E2 + H6N5F1E1 + H5N5E2 + H6N5L2 + H5N5F1E1L1 + H6N5E1L1 + H5N5F1E2 + H6N5E2 + H6N5F1L2 + H6N5F1E1L1 + H6N5F1E2 + H6N5E1L2 + H6N5E2L1 + H6N5E3 + H6N5F1E1L2 + H6N5F1E2L1 + H6N5F1E3 + H6N5F2E1L2 + H6N5F2E2L1 + H7N6E1L2 + H7N6E2L1 + H7N6E3 + H7N6F1E1L2 + H7N6F1E2L1 + H7N6E1L3 + H7N6E2L2 + H7N6E3L1 + H7N6F1E1L3 + H7N6F1E2L2 + H7N6F1E3L1 + H7N6F2E2L2)	0.8195	0.0243	2.96%	0.774131	0.020593	2.66%
**CA3**	Relative abundance of triantennary glycans within complex type glycans	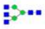	CA3 = (H6N5E1 + H6N5F1E1 + H6N5L2 + H6N5E1L1 + H6N5E2 + H6N5F1L2 + H6N5F1E1L1 + H6N5F1E2 + H6N5E1L2 + H6N5E2L1 + H6N5E3 + H6N5F1E1L2 + H6N5F1E2L1 + H6N5F1E3 + H6N5F2E1L2 + H6N5F2E2L1) / (H3N3E1 + H3N4F1 + H4N4 + H3N5 + H3N3F1E1 + H4N3E1 + H4N4F1 + H5N4 + H3N5F1 + H4N5 + H5N4F1 + H4N4E1 + H4N5F1 + H5N5 + H5N4L1 + H4N4F1E1 + H5N4E1 + H5N5F1 + H4N5E1 + H5N4F1L1 + H5N4F1E1 + H4N5F1E1 + H5N5E1 + H5N4L2 + H5N4E1L1 + H5N4E2 + H5N5F1E1 + H6N5E1 + H5N4F1L2 + H5N4F1E1L1 + H5N4F1E2 + H6N5F1E1 + H5N5E2 + H6N5L2 + H5N5F1E1L1 + H6N5E1L1 + H5N5F1E2 + H6N5E2 + H6N5F1L2 + H6N5F1E1L1 + H6N5F1E2 + H6N5E1L2 + H6N5E2L1 + H6N5E3 + H6N5F1E1L2 + H6N5F1E2L1 + H6N5F1E3 + H6N5F2E1L2 + H6N5F2E2L1 + H7N6E1L2 + H7N6E2L1 + H7N6E3 + H7N6F1E1L2 + H7N6F1E2L1 + H7N6E1L3 + H7N6E2L2 + H7N6E3L1 + H7N6F1E1L3 + H7N6F1E2L2 + H7N6F1E3L1 + H7N6F2E2L2)	0.1616	0.0213	13.16%	0.178731	0.014994	8.39%
**TA2FS0**	Fucosylated, non-sialylated diantennary species within total glycans. Mostly derived from IgG (Clerc et al. 2015).	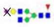	TA2FS0 = (H3N4F1 + H4N4F1 + H3N5F1 + H5N4F1 + H4N5F1 + H5N5F1)	0.0466	0.0101	21.74%	0.063788	0.007242	11.35%
**Fucosylation**									
**A2F**	Fucosylation within diantennary glycans	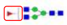	A2F = (H3N4F1 + H4N4F1 + H3N5F1 + H5N4F1 + H4N5F1 + H4N4F1E1 + H5N5F1 + H5N4F1L1 + H5N4F1E1 + H4N5F1E1 + H5N5F1E1 + H5N4F1L2 + H5N4F1E1L1 + H5N4F1E2 + H5N5F1E1L1 + H5N5F1E2) / (H3N4F1 + H4N4 + H3N5 + H4N4F1 + H5N4 + H3N5F1 + H4N5 + H5N4F1 + H4N4E1 + H4N5F1 + H5N5 + H5N4L1 + H4N4F1E1 + H5N4E1 + H5N5F1 + H4N5E1 + H5N4F1L1 + H5N4F1E1 + H4N5F1E1 + H5N5E1 + H5N4L2 + H5N4E1L1 + H5N4E2 + H5N5F1E1 + H5N4F1L2 + H5N4F1E1L1 + H5N4F1E2 + H5N5E2 + H5N5F1E1L1 + H5N5F1E2)	0.1925	0.0201	10.46%	0.205014	0.015817	7.72%
**A4F**	Fucosylation within tetra-antennary glycans	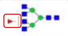	A4F = (H7N6F1E1L2 + H7N6F1E2L1 + H7N6F1E1L3 + H7N6F1E2L2 + H7N6F1E3L1 + H7N6F2E2L2) / (H7N6E1L2 + H7N6E2L1 + H7N6E3 + H7N6F1E1L2 + H7N6F1E2L1 + H7N6E1L3 + H7N6E2L2 + H7N6E3L1 + H7N6F1E1L3 + H7N6F1E2L2 + H7N6F1E3L1 + H7N6F2E2L2)	0.3396	0.0286	8.42%	0.40244	0.02777	6.90%
**A2S0F**	Fucosylation within non-sialylated diantennary glycans	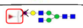	A2S0F = (H3N4F1 + H4N4F1 + H3N5F1 + H5N4F1 + H4N5F1 + H5N5F1) / (H3N4F1 + H4N4 + H3N5 + H4N4F1 + H5N4 + H3N5F1 + H4N5 + H5N4F1 + H4N5F1 + H5N5 + H5N5F1)	0.9334	0.0131	1.41%	0.929363	0.006989	0.75%
**A2EF**	Fucosylation within diantennary glycans with α2,6-linked sialic acid	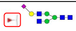	A2EF = (H4N4F1E1 + H5N4F1E1 + H4N5F1E1 + H5N5F1E1 + H5N4F1E1L1 + H5N4F1E2 + H5N5F1E1L1 + H5N5F1E2) / (H4N4E1 + H4N4F1E1 + H5N4E1 + H4N5E1 + H5N4F1E1 + H4N5F1E1 + H5N5E1 + H5N4E1L1 + H5N4E2 + H5N5F1E1 + H5N4F1E1L1 + H5N4F1E2 + H5N5E2 + H5N5F1E1L1 + H5N5F1E2)	0.1302	0.0121	9.30%	0.122182	0.00967	7.91%
**A2E0F**	Fucosylation within diantennary glycans without α2,6-linked sialic acid	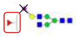	A2E0F = (H3N4F1 + H4N4F1 + H3N5F1 + H5N4F1 + H4N5F1 + H5N5F1 + H5N4F1L1 + H5N4F1L2) / (H3N4F1 + H4N4 + H3N5 + H4N4F1 + H5N4 + H3N5F1 + H4N5 + H5N4F1 + H4N5F1 + H5N5 + H5N4L1 + H5N5F1 + H5N4F1L1 + H5N4L2 + H5N4F1L2)	0.8873	0.0136	1.53%	0.92288	0.006074	0.66%
**A2LF**	Fucosylation within diantennary glycans with α2,3-linked sialic acid	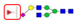	A2LF = (H5N4F1L1 + H5N4F1L2 + H5N4F1E1L1 + H5N5F1E1L1) / (H5N4L1 + H5N4F1L1 + H5N4L2 + H5N4E1L1 + H5N4F1L2 + H5N4F1E1L1 + H5N5F1E1L1)	0.3133	0.0173	5.51%	0.379772	0.013449	3.54%
**A2L0F**	Fucosylation within diantennary glycans without α2,3-linked sialic acid	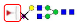	A2L0F = (H3N4F1 + H4N4F1 + H3N5F1 + H5N4F1 + H4N5F1 + H4N4F1E1 + H5N5F1 + H5N4F1E1 + H4N5F1E1 + H5N5F1E1 + H5N4F1E2 + H5N5F1E2) / (H3N4F1 + H4N4 + H3N5 + H4N4F1 + H5N4 + H3N5F1 + H4N5 + H5N4F1 + H4N4E1 + H4N5F1 + H5N5 + H4N4F1E1 + H5N4E1 + H5N5F1 + H4N5E1 + H5N4F1E1 + H4N5F1E1 + H5N5E1 + H5N4E2 + H5N5F1E1 + H5N4F1E2 + H5N5E2 + H5N5F1E2)	0.1788	0.0212	11.83%	0.192445	0.016243	8.44%
**A3F**	Fucosylation within triantennary glycans	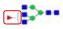	A3F = (H6N5F1E1 + H6N5F1L2 + H6N5F1E1L1 + H6N5F1E2 + H6N5F1E1L2 + H6N5F1E2L1 + H6N5F1E3 + H6N5F2E1L2 + H6N5F2E2L1) / (H6N5E1 + H6N5F1E1 + H6N5L2 + H6N5E1L1 + H6N5E2 + H6N5F1L2 + H6N5F1E1L1 + H6N5F1E2 + H6N5E1L2 + H6N5E2L1 + H6N5E3 + H6N5F1E1L2 + H6N5F1E2L1 + H6N5F1E3 + H6N5F2E1L2 + H6N5F2E2L1)	0.3593	0.0265	7.36%	0.355372	0.016304	4.59%
**A3LF**	Fucosylation within triantennary glycans with α2,3-linked sialic acid	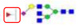	A3LF = (H6N5F1L2 + H6N5F1E1L1 + H6N5F1E1L2 + H6N5F1E2L1 + H6N5F2E1L2 + H6N5F2E2L1) / (H6N5L2 + H6N5E1L1 + H6N5F1L2 + H6N5F1E1L1 + H6N5E1L2 + H6N5E2L1 + H6N5F1E1L2 + H6N5F1E2L1 + H6N5F2E1L2 + H6N5F2E2L1)	0.4326	0.0334	7.71%	0.428352	0.021633	5.05%
**A3L0F**	Fucosylation within triantennary glycans without α2,3-linked sialic acid	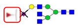	A3L0F = (H6N5F1E1 + H6N5F1E2 + H6N5F1E3) / (H6N5E1 + H6N5F1E1 + H6N5E2 + H6N5F1E2 + H6N5E3 + H6N5F1E3)	0.1487	0.0173	11.65%	0.149752	0.01356	9.05%

**Bisection**									
**A2FSB**	Relative abundance of species with a bisecting GlcNAc within fucosylated sialylated diantennary glycans	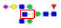	A2FSB = (H4N5F1E1 + H5N5F1E1 + H5N5F1E1L1 + H5N5F1E2) / (H4N4F1E1 + H5N4F1L1 + H5N4F1E1 + H4N5F1E1 + H5N5F1E1 + H5N4F1L2 + H5N4F1E1L1 + H5N4F1E2 + H5N5F1E1L1 + H5N5F1E2)	0.2739	0.0128	4.67%	0.2831	0.014473	5.11%
**A2FS0B**	Relative abundance of species with a bisecting GlcNAc within fucosylated non-sialylated diantennary glycans	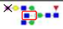	A2FS0B = (H3N5F1 + H4N5F1 + H5N5F1) / (H3N4F1 + H4N4F1 + H3N5F1 + H5N4F1 + H4N5F1 + H5N5F1)	0.1976	0.0160	8.10%	0.195265	0.010292	5.27%
**A2F0SB**	Relative abundance of species with a bisecting GlcNAc within non-fucosylated sialylated diantennary glycans	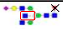	A2F0SB = (H4N5E1 + H5N5E1 + H5N5E2) / (H4N4E1 + H5N4L1 + H5N4E1 + H4N5E1 + H5N5E1 + H5N4L2 + H5N4E1L1 + H5N4E2 + H5N5E2)	0.0120	0.0031	25.79%	0.006848	0.001154	16.86%
**A2F0S0B**	Relative abundance of species with a bisecting GlcNAc within non-fucosylated non-sialylated diantennary glycans	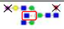	A2F0S0B = (H3N5 + H4N5 + H5N5) / (H4N4 + H3N5 + H5N4 + H4N5 + H5N5)	0.4293	0.0531	12.36%	0.386032	0.034029	8.82%
**Galactosylation per antenna**									
**A2FG**	Galactosylation per antenna within fucosylated diantennary glycans	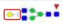	A2FG = (0/2 * (H3N4F1 + H3N5F1) + 1/2 * (H4N4F1 + H4N5F1 + H4N4F1E1 + H4N5F1E1) + 2/2 * (H5N4F1 + H5N5F1 + H5N4F1L1 + H5N4F1E1 + H5N5F1E1 + H5N4F1L2 + H5N4F1E1L1 + H5N4F1E2 + H5N5F1E1L1 + H5N5F1E2)) / (H3N4F1 + H4N4F1 + H3N5F1 + H5N4F1 + H4N5F1 + H4N4F1E1 + H5N5F1 + H5N4F1L1 + H5N4F1E1 + H4N5F1E1 + H5N5F1E1 + H5N4F1L2 + H5N4F1E1L1 + H5N4F1E2 + H5N5F1E1L1 + H5N5F1E2)	0.8476	0.0209	2.46%	0.786626	0.013715	1.74%
**A2F0G**	Galactosylation per antenna within non-fucosylated diantennary glycans	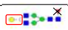	A2F0G = (0/2 * (H3N5) + 1/2 * (H4N4 + H4N5 + H4N4E1 + H4N5E1) + 2/2 * (H5N4 + H5N5 + H5N4L1 + H5N4E1 + H5N5E1 + H5N4L2 + H5N4E1L1 + H5N4E2 + H5N5E2)) / (H4N4 + H3N5 + H5N4 + H4N5 + H4N4E1 + H5N5 + H5N4L1 + H5N4E1 + H4N5E1 + H5N5E1 + H5N4L2 + H5N4E1L1 + H5N4E2 + H5N5E2)	0.9956	0.0011	0.11%	0.998993	0.000251	0.03%
**A2SG**	Galactosylation per antenna within sialylated diantennary glycans	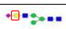	A2SG = (0/2 * (0) + 1/2 * (H4N4E1 + H4N4F1E1 + H4N5E1 + H4N5F1E1) + 2/2 * (H5N4L1 + H5N4E1 + H5N4F1L1 + H5N4F1E1 + H5N5E1 + H5N4L2 + H5N4E1L1 + H5N4E2 + H5N5F1E1 + H5N4F1L2 + H5N4F1E1L1 + H5N4F1E2 + H5N5E2 + H5N5F1E1L1 + H5N5F1E2)) / (H4N4E1 + H5N4L1 + H4N4F1E1 + H5N4E1 + H4N5E1 + H5N4F1L1 + H5N4F1E1 + H4N5F1E1 + H5N5E1 + H5N4L2 + H5N4E1L1 + H5N4E2 + H5N5F1E1 + H5N4F1L2 + H5N4F1E1L1 + H5N4F1E2 + H5N5E2 + H5N5F1E1L1 + H5N5F1E2)	0.9941	0.0011	0.11%	0.99666	0.000444	0.04%
**A2FSG**	Galactosylation per antenna within fucosylated sialylated diantennary glycans	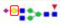	A2FSG = (0/2 * (0) + 1/2 * (H4N4F1E1 + H4N5F1E1) + 2/2 * (H5N4F1L1 + H5N4F1E1 + H5N5F1E1 + H5N4F1L2 + H5N4F1E1L1 + H5N4F1E2 + H5N5F1E1L1 + H5N5F1E2)) / (H4N4F1E1 + H5N4F1L1 + H5N4F1E1 + H4N5F1E1 + H5N5F1E1 + H5N4F1L2 + H5N4F1E1L1 + H5N4F1E2 + H5N5F1E1L1 + H5N5F1E2)	0.9749	0.0043	0.45%	0.974878	0.0021	0.22%
**A2FS0G**	Galactosylation per antenna within fucosylated non-sialylated diantennary glycans	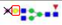	A2FS0G = (0/2 * (H3N4F1 + H3N5F1) + 1/2 * (H4N4F1 + H4N5F1) + 2/2 * (H5N4F1 + H5N5F1)) / (H3N4F1 + H4N4F1 + H3N5F1 + H5N4F1 + H4N5F1 + H5N5F1)	0.5525	0.0218	3.95%	0.517457	0.013041	2.52%
**A2F0S0G**	Galactosylation per antenna within non-fucosylated, non-sialylated diantennary glycans	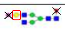	A2F0S0G = (0/2 * (H3N5) + 1/2 * (H4N4 + H4N5) + 2/2 * (H5N4 + H5N5)) / (H4N4 + H3N5 + H5N4 + H4N5 + H5N5)	0.6800	0.0466	6.85%	0.877198	0.014758	1.68%
**Sialylation per antenna**									
**A3S**	Sialylation per antenna within triantennary glycans	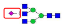	A3S = (0/3 * (0) + 1/3 * (H6N5E1 + H6N5F1E1) + 2/3 * (H6N5L2 + H6N5E1L1 + H6N5E2 + H6N5F1L2 + H6N5F1E1L1 + H6N5F1E2) + 3/3 * (H6N5E1L2 + H6N5E2L1 + H6N5E3 + H6N5F1E1L2 + H6N5F1E2L1 + H6N5F1E3 + H6N5F2E1L2 + H6N5F2E2L1)) / (H6N5E1 + H6N5F1E1 + H6N5L2 + H6N5E1L1 + H6N5E2 + H6N5F1L2 + H6N5F1E1L1 + H6N5F1E2 + H6N5E1L2 + H6N5E2L1 + H6N5E3 + H6N5F1E1L2 + H6N5F1E2L1 + H6N5F1E3 + H6N5F2E1L2 + H6N5F2E2L1)	0.9269	0.0083	0.89%	0.920497	0.008739	0.95%
**A4S**	Sialylation per antenna within tetra-antennary glycans	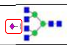	A4S = (0/4 * (0) + 1/4 * (0) + 2/4 * (0) + 3/4 * (H7N6E1L2 + H7N6E2L1 + H7N6E3 + H7N6F1E1L2 + H7N6F1E2L1) + 4/4 * (H7N6E1L3 + H7N6E2L2 + H7N6E3L1 + H7N6F1E1L3 + H7N6F1E2L2 + H7N6F1E3L1 + H7N6F2E2L2)) / (H7N6E1L2 + H7N6E2L1 + H7N6E3 + H7N6F1E1L2 + H7N6F1E2L1 + H7N6E1L3 + H7N6E2L2 + H7N6E3L1 + H7N6F1E1L3 + H7N6F1E2L2 + H7N6F1E3L1 + H7N6F2E2L2)	0.8983	0.0063	0.70%	0.909488	0.008524	0.94%

**Sialylation per galactose**									
**A2GS**	Sialylation per galactose within diantennary glycans		A2GS = ((0/2 * (H3N4F1 + H4N4 + H3N5 + H4N4F1 + H5N4 + H3N5F1 + H4N5 + H5N4F1 + H4N5F1 + H5N5 + H5N5F1) + 1/2 * (H4N4E1 + H5N4L1 + H4N4F1E1 + H5N4E1 + H4N5E1 + H5N4F1L1 + H5N4F1E1 + H4N5F1E1 + H5N5E1 + H5N5F1E1) + 2/2 * (H5N4L2 + H5N4E1L1 + H5N4E2 + H5N4F1L2 + H5N4F1E1L1 + H5N4F1E2 + H5N5E2 + H5N5F1E1L1 + H5N5F1E2)) / (H3N4F1 + H4N4 + H3N5 + H4N4F1 + H5N4 + H3N5F1 + H4N5 + H5N4F1 + H4N4E1 + H4N5F1 + H5N5 + H5N4L1 + H4N4F1E1 + H5N4E1 + H5N5F1 + H4N5E1 + H5N4F1L1 + H5N4F1E1 + H4N5F1E1 + H5N5E1 + H5N4L2 + H5N4E1L1 + H5N4E2 + H5N5F1E1 + H5N4F1L2 + H5N4F1E1L1 + H5N4F1E2 + H5N5E2 + H5N5F1E1L1 + H5N5F1E2)) / ((0/2 * (H3N4F1 + H3N5 + H3N5F1) + 1/2 * (H4N4 + H4N4F1 + H4N5 + H4N4E1 + H4N5F1 + H4N4F1E1 + H4N5E1 + H4N5F1E1) + 2/2 * (H5N4 + H5N4F1 + H5N5 + H5N4L1 + H5N4E1 + H5N5F1 + H5N4F1L1 + H5N4F1E1 + H5N5E1 + H5N4L2 + H5N4E1L1 + H5N4E2 + H5N5F1E1 + H5N4F1L2 + H5N4F1E1L1 + H5N4F1E2 + H5N5E2 + H5N5F1E1L1 + H5N5F1E2)) / (H3N4F1 + H4N4 + H3N5 + H4N4F1 + H5N4 + H3N5F1 + H4N5 + H5N4F1 + H4N4E1 + H4N5F1 + H5N5 + H5N4L1 + H4N4F1E1 + H5N4E1 + H5N5F1 + H4N5E1 + H5N4F1L1 + H5N4F1E1 + H4N5F1E1 + H5N5E1 + H5N4L2 + H5N4E1L1 + H5N4E2 + H5N5F1E1 + H5N4F1L2 + H5N4F1E1L1 + H5N4F1E2 + H5N5E2 + H5N5F1E1L1 + H5N5F1E2))	0.8924	0.0144	1.61%	0.864516	0.010983	1.27%
**A2FGS**	Sialylation per galactose within fucosylated diantennary glycans	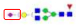	A2FGS = ((0/2 * (H3N4F1 + H4N4F1 + H3N5F1 + H5N4F1 + H4N5F1 + H5N5F1) + 1/2 * (H4N4F1E1 + H5N4F1L1 + H5N4F1E1 + H4N5F1E1 + H5N5F1E1) + 2/2 * (H5N4F1L2 + H5N4F1E1L1 + H5N4F1E2 + H5N5F1E1L1 + H5N5F1E2)) / (H3N4F1 + H4N4F1 + H3N5F1 + H5N4F1 + H4N5F1 + H4N4F1E1 + H5N5F1 + H5N4F1L1 + H5N4F1E1 + H4N5F1E1 + H5N5F1E1 + H5N4F1L2 + H5N4F1E1L1 + H5N4F1E2 + H5N5F1E1L1 + H5N5F1E2)) / ((0/2 * (H3N4F1 + H3N5F1) + 1/2 * (H4N4F1 + H4N5F1 + H4N4F1E1 + H4N5F1E1) + 2/2 * (H5N4F1 + H5N5F1 + H5N4F1L1 + H5N4F1E1 + H5N5F1E1 + H5N4F1L2 + H5N4F1E1L1 + H5N4F1E2 + H5N5F1E1L1 + H5N5F1E2)) / (H3N4F1 + H4N4F1 + H3N5F1 + H5N4F1 + H4N5F1 + H4N4F1E1 + H5N5F1 + H5N4F1L1 + H5N4F1E1 + H4N5F1E1 + H5N5F1E1 + H5N4F1L2 + H5N4F1E1L1 + H5N4F1E2 + H5N5F1E1L1 + H5N5F1E2))	0.6681	0.0340	5.09%	0.603623	0.021313	3.53%
**A2F0GS**	Sialylation per galactose within non-fucosylated diantennary glycans	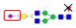	A2F0GS = ((0/2 * (H4N4 + H3N5 + H5N4 + H4N5 + H5N5) + 1/2 * (H4N4E1 + H5N4L1 + H5N4E1 + H4N5E1 + H5N5E1) + 2/2 * (H5N4L2 + H5N4E1L1 + H5N4E2 + H5N5E2)) / (H4N4 + H3N5 + H5N4 + H4N5 + H4N4E1 + H5N5 + H5N4L1 + H5N4E1 + H4N5E1 + H5N5E1 + H5N4L2 + H5N4E1L1 + H5N4E2 + H5N5E2)) / ((0/2 * (H3N5) + 1/2 * (H4N4 + H4N5 + H4N4E1 + H4N5E1) + 2/2 * (H5N4 + H5N5 + H5N4L1 + H5N4E1 + H5N5E1 + H5N4L2 + H5N4E1L1 + H5N4E2 + H5N5E2)) / (H4N4 + H3N5 + H5N4 + H4N5 + H4N4E1 + H5N5 + H5N4L1 + H5N4E1 + H4N5E1 + H5N5E1 + H5N4L2 + H5N4E1L1 + H5N4E2 + H5N5E2))	0.9383	0.0062	0.66%	0.916688	0.008209	0.90%
**A4FGS**	Sialylation per galactose within fucosylated tetra-antennary glycans	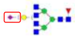	A4FGS = ((0/4 * (0) + 1/4 * (0) + 2/4 * (0) + 3/4 * (H7N6F1E1L2 + H7N6F1E2L1) + 4/4 * (H7N6F1E1L3 + H7N6F1E2L2 + H7N6F1E3L1 + H7N6F2E2L2)) / (H7N6E1L2 + H7N6E2L1 + H7N6E3 + H7N6F1E1L2 + H7N6F1E2L1 + H7N6E1L3 + H7N6E2L2 + H7N6E3L1 + H7N6F1E1L3 + H7N6F1E2L2 + H7N6F1E3L1 + H7N6F2E2L2)) / ((0/4 * (0) + 1/4 * (0) + 2/4 * (0) + 3/4 * (0) + 4/4 * (H7N6F1E1L2 + H7N6F1E2L1 + H7N6F1E1L3 + H7N6F1E2L2 + H7N6F1E3L1 + H7N6F2E2L2)) / (H7N6E1L2 + H7N6E2L1 + H7N6E3 + H7N6F1E1L2 + H7N6F1E2L1 + H7N6E1L3 + H7N6E2L2 + H7N6E3L1 + H7N6F1E1L3 + H7N6F1E2L2 + H7N6F1E3L1 + H7N6F2E2L2))	0.9084	0.0091	1.00%	0.930707	0.008443	0.91%
**α2,3-sialylation per antenna**									
**A2L**	α2,3-sialylation per antenna within diantennary glycans	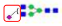	A2L = (0/2 * (H3N4F1 + H4N4 + H3N5 + H4N4F1 + H5N4 + H3N5F1 + H4N5 + H5N4F1 + H4N4E1 + H4N5F1 + H5N5 + H4N4F1E1 + H5N4E1 + H5N5F1 + H4N5E1 + H5N4F1E1 + H4N5F1E1 + H5N5E1 + H5N4E2 + H5N5F1E1 + H5N4F1E2 + H5N5E2 + H5N5F1E2) + 1/2 * (H5N4L1 + H5N4F1L1 + H5N4E1L1 + H5N4F1E1L1 + H5N5F1E1L1) + 2/2 * (H5N4L2 + H5N4F1L2)) / (H3N4F1 + H4N4 + H3N5 + H4N4F1 + H5N4 + H3N5F1 + H4N5 + H5N4F1 + H4N4E1 + H4N5F1 + H5N5 + H5N4L1 + H4N4F1E1 + H5N4E1 + H5N5F1 + H4N5E1 + H5N4F1L1 + H5N4F1E1 + H4N5F1E1 + H5N5E1 + H5N4L2 + H5N4E1L1 + H5N4E2 + H5N5F1E1 + H5N4F1L2 + H5N4F1E1L1 + H5N4F1E2 + H5N5E2 + H5N5F1E1L1 + H5N5F1E2)	0.0568	0.0044	7.70%	0.036526	0.003373	9.23%
**A3L**	α2,3-sialylation per antenna within triantennary glycans	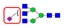	A3L = (0/3 * (H6N5E1 + H6N5F1E1 + H6N5E2 + H6N5F1E2 + H6N5E3 + H6N5F1E3) + 1/3 * (H6N5E1L1 + H6N5F1E1L1 + H6N5E2L1 + H6N5F1E2L1 + H6N5F2E2L1) + 2/3 * (H6N5L2 + H6N5F1L2 + H6N5E1L2 + H6N5F1E1L2 + H6N5F2E1L2) + 3/3 * (0)) / (H6N5E1 + H6N5F1E1 + H6N5L2 + H6N5E1L1 + H6N5E2 + H6N5F1L2 + H6N5F1E1L1 + H6N5F1E2 + H6N5E1L2 + H6N5E2L1 + H6N5E3 + H6N5F1E1L2 + H6N5F1E2L1 + H6N5F1E3 + H6N5F2E1L2 + H6N5F2E2L1)	0.2779	0.0052	1.86%	0.269443	0.007864	2.92%
**A4L**	α2,3-sialylation per antenna within tetra-antennary glycans	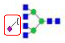	A4L = (0/4 * (H7N6E3) + 1/4 * (H7N6E2L1 + H7N6F1E2L1 + H7N6E3L1 + H7N6F1E3L1) + 2/4 * (H7N6E1L2 + H7N6F1E1L2 + H7N6E2L2 + H7N6F1E2L2 + H7N6F2E2L2) + 3/4 * (H7N6E1L3 + H7N6F1E1L3) + 4/4 * (0)) / (H7N6E1L2 + H7N6E2L1 + H7N6E3 + H7N6F1E1L2 + H7N6F1E2L1 + H7N6E1L3 + H7N6E2L2 + H7N6E3L1 + H7N6F1E1L3 + H7N6F1E2L2 + H7N6F1E3L1 + H7N6F2E2L2)	0.4712	0.0103	2.18%	0.515143	0.01139	2.21%

**α2,6-sialylation per antenna**									
**A2E**	α2,6-sialylation per antenna within diantennary glycans	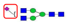	A2E = (0/2 * (H3N4F1 + H4N4 + H3N5 + H4N4F1 + H5N4 + H3N5F1 + H4N5 + H5N4F1 + H4N5F1 + H5N5 + H5N4L1 + H5N5F1 + H5N4F1L1 + H5N4L2 + H5N4F1L2) + 1/2 * (H4N4E1 + H4N4F1E1 + H5N4E1 + H4N5E1 + H5N4F1E1 + H4N5F1E1 + H5N5E1 + H5N4E1L1 + H5N5F1E1 + H5N4F1E1L1 + H5N5F1E1L1) + 2/2 * (H5N4E2 + H5N4F1E2 + H5N5E2 + H5N5F1E2)) / (H3N4F1 + H4N4 + H3N5 + H4N4F1 + H5N4 + H3N5F1 + H4N5 + H5N4F1 + H4N4E1 + H4N5F1 + H5N5 + H5N4L1 + H4N4F1E1 + H5N4E1 + H5N5F1 + H4N5E1 + H5N4F1L1 + H5N4F1E1 + H4N5F1E1 + H5N5E1 + H5N4L2 + H5N4E1L1 + H5N4E2 + H5N5F1E1 + H5N4F1L2 + H5N4F1E1L1 + H5N4F1E2 + H5N5E2 + H5N5F1E1L1 + H5N5F1E2)	0.8062	0.0200	2.47%	0.789328	0.015944	2.02%
**A3E**	α2,6-sialylation per antenna within triantennary glycans	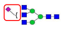	A3E = (0/3 * (H6N5L2 + H6N5F1L2) + 1/3 * (H6N5E1 + H6N5F1E1 + H6N5E1L1 + H6N5F1E1L1 + H6N5E1L2 + H6N5F1E1L2 + H6N5F2E1L2) + 2/3 * (H6N5E2 + H6N5F1E2 + H6N5E2L1 + H6N5F1E2L1 + H6N5F2E2L1) + 3/3 * (H6N5E3 + H6N5F1E3)) / (H6N5E1 + H6N5F1E1 + H6N5L2 + H6N5E1L1 + H6N5E2 + H6N5F1L2 + H6N5F1E1L1 + H6N5F1E2 + H6N5E1L2 + H6N5E2L1 + H6N5E3 + H6N5F1E1L2 + H6N5F1E2L1 + H6N5F1E3 + H6N5F2E1L2 + H6N5F2E2L1)	0.6490	0.0072	1.11%	0.651003	0.006438	0.99%
**A4E**	α2,6-sialylation per antenna within tetra-antennary glycans	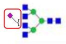	A4E = (0/4 * (0) + 1/4 * (H7N6E1L2 + H7N6F1E1L2 + H7N6E1L3 + H7N6F1E1L3) + 2/4 * (H7N6E2L1 + H7N6F1E2L1 + H7N6E2L2 + H7N6F1E2L2 + H7N6F2E2L2) + 3/4 * (H7N6E3 + H7N6E3L1 + H7N6F1E3L1) + 4/4 * (0)) / (H7N6E1L2 + H7N6E2L1 + H7N6E3 + H7N6F1E1L2 + H7N6F1E2L1 + H7N6E1L3 + H7N6E2L2 + H7N6E3L1 + H7N6F1E1L3 + H7N6F1E2L2 + H7N6F1E3L1 + H7N6F2E2L2)	0.4272	0.0082	1.93%	0.39385	0.011386	2.89%
**α2,3-sialylation per galactose**									
**A2FGL**	α2,3-sialylation per galactose within fucosylated diantennary glycans	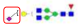	A2FGL = ((0/2 * (H3N4F1 + H4N4F1 + H3N5F1 + H5N4F1 + H4N5F1 + H4N4F1E1 + H5N5F1 + H5N4F1E1 + H4N5F1E1 + H5N5F1E1 + H5N4F1E2 + H5N5F1E2) + 1/2 * (H5N4F1L1 + H5N4F1E1L1 + H5N5F1E1L1) + 2/2 * (H5N4F1L2)) / (H3N4F1 + H4N4F1 + H3N5F1 + H5N4F1 + H4N5F1 + H4N4F1E1 + H5N5F1 + H5N4F1L1 + H5N4F1E1 + H4N5F1E1 + H5N5F1E1 + H5N4F1L2 + H5N4F1E1L1 + H5N4F1E2 + H5N5F1E1L1 + H5N5F1E2)) / ((0/2 * (H3N4F1 + H3N5F1) + 1/2 * (H4N4F1 + H4N5F1 + H4N4F1E1 + H4N5F1E1) + 2/2 * (H5N4F1 + H5N5F1 + H5N4F1L1 + H5N4F1E1 + H5N5F1E1 + H5N4F1L2 + H5N4F1E1L1 + H5N4F1E2 + H5N5F1E1L1 + H5N5F1E2)) / (H3N4F1 + H4N4F1 + H3N5F1 + H5N4F1 + H4N5F1 + H4N4F1E1 + H5N5F1 + H5N4F1L1 + H5N4F1E1 + H4N5F1E1 + H5N5F1E1 + H5N4F1L2 + H5N4F1E1L1 + H5N4F1E2 + H5N5F1E1L1 + H5N5F1E2))	0.1278	0.0101	7.88%	0.092623	0.006654	7.18%
**A2F0GL**	α2,3-sialylation per galactose within non-fucosylated diantennary glycans	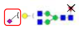	A2F0GL = ((0/2 * (H4N4 + H3N5 + H5N4 + H4N5 + H4N4E1 + H5N5 + H5N4E1 + H4N5E1 + H5N5E1 + H5N4E2 + H5N5E2) + 1/2 * (H5N4L1 + H5N4E1L1) + 2/2 * (H5N4L2)) / (H4N4 + H3N5 + H5N4 + H4N5 + H4N4E1 + H5N5 + H5N4L1 + H5N4E1 + H4N5E1 + H5N5E1 + H5N4L2 + H5N4E1L1 + H5N4E2 + H5N5E2)) / ((0/2 * (H3N5) + 1/2 * (H4N4 + H4N5 + H4N4E1 + H4N5E1) + 2/2 * (H5N4 + H5N5 + H5N4L1 + H5N4E1 + H5N5E1 + H5N4L2 + H5N4E1L1 + H5N4E2 + H5N5E2)) / (H4N4 + H3N5 + H5N4 + H4N5 + H4N4E1 + H5N5 + H5N4L1 + H5N4E1 + H4N5E1 + H5N5E1 + H5N4L2 + H5N4E1L1 + H5N4E2 + H5N5E2))	0.0445	0.0039	8.67%	0.027291	0.002993	10.97%
**A4FGL**	α2,3-sialylation per galactose within fucosylated tetra-antennary glycans	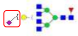	A4FGL = ((0/4 * (0) + 1/4 * (H7N6F1E2L1 + H7N6F1E3L1) + 2/4 * (H7N6F1E1L2 + H7N6F1E2L2 + H7N6F2E2L2) + 3/4 * (H7N6F1E1L3) + 4/4 * (0)) / (H7N6E1L2 + H7N6E2L1 + H7N6E3 + H7N6F1E1L2 + H7N6F1E2L1 + H7N6E1L3 + H7N6E2L2 + H7N6E3L1 + H7N6F1E1L3 + H7N6F1E2L2 + H7N6F1E3L1 + H7N6F2E2L2)) / ((0/4 * (0) + 1/4 * (0) + 2/4 * (0) + 3/4 * (0) + 4/4 * (H7N6F1E1L2 + H7N6F1E2L1 + H7N6F1E1L3 + H7N6F1E2L2 + H7N6F1E3L1 + H7N6F2E2L2)) / (H7N6E1L2 + H7N6E2L1 + H7N6E3 + H7N6F1E1L2 + H7N6F1E2L1 + H7N6E1L3 + H7N6E2L2 + H7N6E3L1 + H7N6F1E1L3 + H7N6F1E2L2 + H7N6F1E3L1 + H7N6F2E2L2))	0.4869	0.0142	2.92%	0.554988	0.0148	2.67%
**A4F0GL**	α2,3-sialylation per galactose within non-fucosylated tetra-antennary glycans	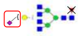	A4F0GL = ((0/4 * (H7N6E3) + 1/4 * (H7N6E2L1 + H7N6E3L1) + 2/4 * (H7N6E1L2 + H7N6E2L2) + 3/4 * (H7N6E1L3) + 4/4 * (0)) / (H7N6E1L2 + H7N6E2L1 + H7N6E3 + H7N6F1E1L2 + H7N6F1E2L1 + H7N6E1L3 + H7N6E2L2 + H7N6E3L1 + H7N6F1E1L3 + H7N6F1E2L2 + H7N6F1E3L1 + H7N6F2E2L2)) / ((0/4 * (0) + 1/4 * (0) + 2/4 * (0) + 3/4 * (0) + 4/4 * (H7N6E1L2 + H7N6E2L1 + H7N6E3 + H7N6E1L3 + H7N6E2L2 + H7N6E3L1)) / (H7N6E1L2 + H7N6E2L1 + H7N6E3 + H7N6F1E1L2 + H7N6F1E2L1 + H7N6E1L3 + H7N6E2L2 + H7N6E3L1 + H7N6F1E1L3 + H7N6F1E2L2 + H7N6F1E3L1 + H7N6F2E2L2))	0.4629	0.0120	2.58%	0.488436	0.013871	2.84%
**α2,6-sialylation per galactose**									
**A2FGE**	α2,6-sialylation per galactose within fucosylated diantennary glycans	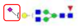	A2FGE = ((0/2 * (H3N4F1 + H4N4F1 + H3N5F1 + H5N4F1 + H4N5F1 + H5N5F1 + H5N4F1L1 + H5N4F1L2) + 1/2 * (H4N4F1E1 + H5N4F1E1 + H4N5F1E1 + H5N5F1E1 + H5N4F1E1L1 + H5N5F1E1L1) + 2/2 * (H5N4F1E2 + H5N5F1E2)) / (H3N4F1 + H4N4F1 + H3N5F1 + H5N4F1 + H4N5F1 + H4N4F1E1 + H5N5F1 + H5N4F1L1 + H5N4F1E1 + H4N5F1E1 + H5N5F1E1 + H5N4F1L2 + H5N4F1E1L1 + H5N4F1E2 + H5N5F1E1L1 + H5N5F1E2)) / ((0/2 * (H3N4F1 + H3N5F1) + 1/2 * (H4N4F1 + H4N5F1 + H4N4F1E1 + H4N5F1E1) + 2/2 * (H5N4F1 + H5N5F1 + H5N4F1L1 + H5N4F1E1 + H5N5F1E1 + H5N4F1L2 + H5N4F1E1L1 + H5N4F1E2 + H5N5F1E1L1 + H5N5F1E2)) / (H3N4F1 + H4N4F1 + H3N5F1 + H5N4F1 + H4N5F1 + H4N4F1E1 + H5N5F1 + H5N4F1L1 + H5N4F1E1 + H4N5F1E1 + H5N5F1E1 + H5N4F1L2 + H5N4F1E1L1 + H5N4F1E2 + H5N5F1E1L1 + H5N5F1E2))	0.5375	0.0269	5.00%	0.51117	0.017797	3.48%
**A2F0GE**	α2,6-sialylation per galactose within non-fucosylated diantennary glycans	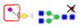	A2F0GE = ((0/2 * (H4N4 + H3N5 + H5N4 + H4N5 + H5N5 + H5N4L1 + H5N4L2) + 1/2 * (H4N4E1 + H5N4E1 + H4N5E1 + H5N5E1 + H5N4E1L1) + 2/2 * (H5N4E2 + H5N5E2)) / (H4N4 + H3N5 + H5N4 + H4N5 + H4N4E1 + H5N5 + H5N4L1 + H5N4E1 + H4N5E1 + H5N5E1 + H5N4L2 + H5N4E1L1 + H5N4E2 + H5N5E2)) / ((0/2 * (H3N5) + 1/2 * (H4N4 + H4N5 + H4N4E1 + H4N5E1) + 2/2 * (H5N4 + H5N5 + H5N4L1 + H5N4E1 + H5N5E1 + H5N4L2 + H5N4E1L1 + H5N4E2 + H5N5E2)) / (H4N4 + H3N5 + H5N4 + H4N5 + H4N4E1 + H5N5 + H5N4L1 + H5N4E1 + H4N5E1 + H5N5E1 + H5N4L2 + H5N4E1L1 + H5N4E2 + H5N5E2))	0.8937	0.0085	0.95%	0.889369	0.009482	1.07%
**A4FGE**	α2,6-sialylation per galactose within fucosylated tetra-antennary glycans	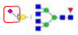	A4FGE = ((0/4 * (0) + 1/4 * (H7N6F1E1L2 + H7N6F1E1L3) + 2/4 * (H7N6F1E2L1 + H7N6F1E2L2 + H7N6F2E2L2) + 3/4 * (H7N6F1E3L1) + 4/4 * (0)) / (H7N6E1L2 + H7N6E2L1 + H7N6E3 + H7N6F1E1L2 + H7N6F1E2L1 + H7N6E1L3 + H7N6E2L2 + H7N6E3L1 + H7N6F1E1L3 + H7N6F1E2L2 + H7N6F1E3L1 + H7N6F2E2L2)) / ((0/4 * (0) + 1/4 * (0) + 2/4 * (0) + 3/4 * (0) + 4/4 * (H7N6F1E1L2 + H7N6F1E2L1 + H7N6F1E1L3 + H7N6F1E2L2 + H7N6F1E3L1 + H7N6F2E2L2)) / (H7N6E1L2 + H7N6E2L1 + H7N6E3 + H7N6F1E1L2 + H7N6F1E2L1 + H7N6E1L3 + H7N6E2L2 + H7N6E3L1 + H7N6F1E1L3 + H7N6F1E2L2 + H7N6F1E3L1 + H7N6F2E2L2))	0.4216	0.0114	2.70%	0.375431	0.009097	2.42%
**A4F0GE**	α2,6-sialylation per galactose within non-fucosylated tetra-antennary glycans	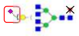	A4F0GE = ((0/4 * (0) + 1/4 * (H7N6E1L2 + H7N6E1L3) + 2/4 * (H7N6E2L1 + H7N6E2L2) + 3/4 * (H7N6E3 + H7N6E3L1) + 4/4 * (0)) / (H7N6E1L2 + H7N6E2L1 + H7N6E3 + H7N6F1E1L2 + H7N6F1E2L1 + H7N6E1L3 + H7N6E2L2 + H7N6E3L1 + H7N6F1E1L3 + H7N6F1E2L2 + H7N6F1E3L1 + H7N6F2E2L2)) / ((0/4 * (0) + 1/4 * (0) + 2/4 * (0) + 3/4 * (0) + 4/4 * (H7N6E1L2 + H7N6E2L1 + H7N6E3 + H7N6E1L3 + H7N6E2L2 + H7N6E3L1)) / (H7N6E1L2 + H7N6E2L1 + H7N6E3 + H7N6F1E1L2 + H7N6F1E2L1 + H7N6E1L3 + H7N6E2L2 + H7N6E3L1 + H7N6F1E1L3 + H7N6F1E2L2 + H7N6F1E3L1 + H7N6F2E2L2))	0.4305	0.0103	2.38%	0.406343	0.017863	4.40%

Supplementary Table 4. DiaGene Imputed clinical characteristics

Name	DiaGene with imputed data	Count of imputations
Duration of diabetes, yr, median (IQR)	7.90 (3.40-14.00)	116
BMI, kg/m2, mean (±sd)	30.45 (5.45)	126
HDL-c, mmol/L, mean (±sd)	1.17 (0.32)	90
Non-HDL-c, mmol/L, mean (±sd)	3.12 (0.90)	90
Creatinine, µmol/L, mean (±sd)	83.31 (28.24)	118
HbA1c, mmol/mol, mean (±sd)	53.23 (1.06)	89
Systolic blood pressure, mmHg, mean (±sd)	142.38 (18.97)	123
Smoking, no/former/yes	479/1014/322	172

Supplementary Table 5. Hoorn DCS Imputed clinical characteristics

Name	Hoorn DCS with imputed data	Count of imputations
Duration of diabetes, yr, median (IQR)	5.96 (2.92-9.90)	1
BMI, kg/m2, mean (±sd)	30.31 (5.43)	19
HDL-c, mmol/L, mean (±sd)	1.18 (0.39)	19
Non-HDL-c, mmol/L, mean (±sd)	3.46 (1.47)	20
Creatinine, µmol/L, mean (±sd)	79.75 (22.17)	18
HbA1c, mmol/mol, mean (±sd)	50.84 (11.44)	18
Systolic blood pressure, mmHg, mean (±sd)	143.80 (19.29)	9
Smoking, no/former/yes	920/335/263	13

Supplementary Table 6. Meta analysed associations between derived glycan traits and cardiovascular disease in type 2 diabetes in the basic model. Glycan values are centered and scaled. The *p*-values after FDR correction that are significant (≤0.05) are highlighted in green. Red represents a positive association and blue a negative association. Basic model: age, sex and the interaction thereof.

Prevalent (cross-sectional)										
**glycome**	**OR**	**SE**	**CIlower**	**CIupper**	**pval**	**I2**	**I2pval**	**n**	**nevent**	**pval.FDR**
A2E	1.0888	0.0425	1.0018	1.1833	0.0452	0.0000	0.9965	3187	916	0.1043
A2E0F	0.8581	0.0423	0.7898	0.9323	0.0003	0.0000	0.6188	3187	916	0.0022
A2EF	0.9101	0.0425	0.8373	0.9892	0.0267	0.0000	0.7852	3187	916	0.0752
A2F	0.9076	0.0424	0.8353	0.9862	0.0221	0.0000	0.8689	3187	916	0.0710
A2F0G	0.9725	0.0417	0.8961	1.0555	0.5048	0.0000	0.3700	3187	916	0.6883
A2F0GE	1.0391	0.0433	0.9546	1.1311	0.3753	0.0000	0.6857	3187	916	0.5629
A2F0GL	0.9446	0.0616	0.8371	1.0659	0.3553	0.4894	0.1617	3187	916	0.5514
A2F0GS	1.0043	0.0431	0.9228	1.0929	0.9216	0.0000	0.6720	3187	916	0.9644
A2F0S0B	1.1318	0.0726	0.9818	1.3047	0.0880	0.6338	0.0985	3187	916	0.1799
A2F0S0G	0.9265	0.0425	0.8524	1.0070	0.0725	0.0000	0.8385	3187	916	0.1553
A2F0SB	0.9907	0.0415	0.9133	1.0746	0.8211	0.0000	0.6122	3187	916	0.9032
A2FG	0.9921	0.0434	0.9112	1.0802	0.8553	0.0000	0.9302	3187	916	0.9164
A2FGE	1.0527	0.0426	0.9684	1.1442	0.2278	0.0000	0.7157	3187	916	0.3701
A2FGL	1.1097	0.0723	0.9632	1.2786	0.1496	0.6396	0.0958	3187	916	0.2806
A2FGS	1.1170	0.0430	1.0267	1.2153	0.0101	0.0000	0.5760	3187	916	0.0413
A2FS0B	1.3827	0.0444	1.2674	1.5084	0.0000	0.0000	0.8936	3187	916	0.0000
A2FS0G	0.8895	0.0475	0.8104	0.9764	0.0138	0.0000	0.6219	3187	916	0.0494
A2FSB	1.0139	0.0442	0.9297	1.1057	0.7556	0.0760	0.2982	3187	916	0.9032
A2FSG	1.0001	0.0424	0.9203	1.0868	0.9987	0.0000	0.4300	3187	916	0.9987
A2GS	1.1000	0.0429	1.0114	1.1964	0.0261	0.0000	0.9569	3187	916	0.0752
A2L	1.0025	0.0426	0.9222	1.0899	0.9525	0.0000	0.9964	3187	916	0.9741
A2L0F	0.9014	0.0424	0.8295	0.9794	0.0143	0.0000	0.6431	3187	916	0.0494
A2LF	1.0252	0.1111	0.8245	1.2746	0.8229	0.8402	0.0124	3187	916	0.9032
A2S0F	0.8808	0.0415	0.8120	0.9554	0.0022	0.0000	0.6803	3187	916	0.0125
A2SG	1.0643	0.0422	0.9799	1.1560	0.1395	0.0000	0.7530	3187	916	0.2729
A3E	1.1687	0.0427	1.0748	1.2708	0.0003	0.0000	0.3510	3187	916	0.0022
A3F	0.9469	0.1206	0.7477	1.1994	0.6512	0.8599	0.0075	3187	916	0.8618
A3L	0.8696	0.0517	0.7858	0.9623	0.0069	0.3114	0.2282	3187	916	0.0343
A3L0F	0.8562	0.1139	0.6849	1.0703	0.1727	0.8363	0.0135	3187	916	0.3109
A3LF	0.9709	0.1187	0.7693	1.2253	0.8035	0.8569	0.0082	3187	916	0.9032
A3S	0.9654	0.0418	0.8895	1.0478	0.4000	0.0000	0.7322	3187	916	0.5807
A4E	1.2270	0.0509	1.1106	1.3556	0.0001	0.2699	0.2419	3187	916	0.0009
A4F	0.9736	0.1154	0.7765	1.2207	0.8167	0.8473	0.0105	3187	916	0.9032
A4F0GE	1.2457	0.0424	1.1464	1.3537	0.0000	0.0000	0.6093	3187	916	0.0000
A4F0GL	0.8266	0.0515	0.7471	0.9144	0.0002	0.2996	0.2321	3187	916	0.0022
A4FGE	1.0781	0.1076	0.8731	1.3311	0.4847	0.8372	0.0132	3187	916	0.6816
A4FGL	0.8874	0.0996	0.7300	1.0787	0.2303	0.8087	0.0222	3187	916	0.3701
A4FGS	0.8943	0.0427	0.8226	0.9723	0.0088	0.0000	0.5527	3187	916	0.0397
A4L	0.8222	0.0637	0.7257	0.9315	0.0021	0.5266	0.1461	3187	916	0.0125
A4S	0.9290	0.0571	0.8306	1.0391	0.1974	0.4322	0.1845	3187	916	0.3417
CA2	0.9171	0.0434	0.8423	0.9986	0.0464	0.0000	0.7137	3187	916	0.1043
CA3	1.0931	0.0437	1.0034	1.1907	0.0415	0.0000	0.7853	3187	916	0.1037
MHy	1.0130	0.0438	0.9296	1.1038	0.7689	0.0000	0.5426	3187	916	0.9032
MM	0.9834	0.0417	0.9062	1.0673	0.6891	0.0000	0.4099	3187	916	0.8859
TA2FS0	0.9125	0.0433	0.8383	0.9933	0.0344	0.0000	0.9959	3187	916	0.0910

Supplementary Table 6. Meta analysed associations between derived glycan traits and cardiovascular disease in type 2 diabetes in the basic model. Glycan values are centered and scaled. The *p*-values after FDR correction that are significant (≤0.05) are highlighted in green. Red represents a positive association and blue a negative association. Basic model: age, sex and the interaction thereof.

	Incident (Cox)									
**glycome**	**HR**	**SE**	**CIlower**	**CIupper**	**pval**	**I2**	**I2pval**	**n**	**nevent**	**pval.FDR**
A2E	1.1656	0.1648	0.8439	1.6101	0.3523	0.7876	0.0300	2254	201	0.7179
A2E0F	0.9380	0.0714	0.8156	1.0789	0.3704	0.0000	0.3523	2254	201	0.7179
A2EF	0.9894	0.0699	0.8627	1.1346	0.8784	0.0000	0.8879	2254	201	0.9469
A2F	0.9475	0.0727	0.8217	1.0926	0.4582	0.0000	0.6274	2254	201	0.7364
A2F0G	1.1315	0.0782	0.9708	1.3188	0.1140	0.0000	0.5023	2254	201	0.6264
A2F0GE	1.0665	0.0930	0.8888	1.2797	0.4890	0.3775	0.2050	2254	201	0.7516
A2F0GL	0.9560	0.0728	0.8288	1.1027	0.5364	0.0000	0.9739	2254	201	0.7516
A2F0GS	1.0429	0.1094	0.8416	1.2924	0.7011	0.5594	0.1319	2254	201	0.8303
A2F0S0B	0.9375	0.0781	0.8045	1.0926	0.4088	0.1303	0.2836	2254	201	0.7179
A2F0S0G	1.0954	0.0728	0.9497	1.2633	0.2109	0.0000	0.5852	2254	201	0.6264
A2F0SB	0.9419	0.0760	0.8115	1.0932	0.4307	0.0000	0.3863	2254	201	0.7179
A2FG	1.0586	0.0955	0.8779	1.2765	0.5510	0.3640	0.2099	2254	201	0.7516
A2FGE	1.1404	0.1508	0.8485	1.5326	0.3837	0.7688	0.0375	2254	201	0.7179
A2FGL	1.1071	0.0705	0.9643	1.2711	0.1488	0.0000	0.7981	2254	201	0.6264
A2FGS	1.2037	0.1452	0.9056	1.6000	0.2016	0.7447	0.0478	2254	201	0.6264
A2FS0B	1.0119	0.1593	0.7405	1.3829	0.9406	0.7909	0.0288	2254	201	0.9469
A2FS0G	0.8934	0.0816	0.7613	1.0484	0.1674	0.0000	0.6576	2254	201	0.6264
A2FSB	0.9332	0.0740	0.8073	1.0788	0.3501	0.0000	0.4234	2254	201	0.7179
A2FSG	1.1744	0.1056	0.9548	1.4445	0.1281	0.4624	0.1726	2254	201	0.6264
A2GS	1.1708	0.1502	0.8722	1.5715	0.2938	0.7467	0.0469	2254	201	0.7179
A2L	1.0332	0.0715	0.8982	1.1886	0.6476	0.0000	0.5183	2254	201	0.8079
A2L0F	0.9365	0.0725	0.8125	1.0795	0.3653	0.0000	0.7313	2254	201	0.7179
A2LF	1.0917	0.0951	0.9061	1.3155	0.3560	0.3808	0.2038	2254	201	0.7179
A2S0F	0.9563	0.0704	0.8330	1.0978	0.5253	0.0000	0.4107	2254	201	0.7516
A2SG	1.1079	0.0791	0.9487	1.2938	0.1954	0.0000	0.5048	2254	201	0.6264
A3E	1.1436	0.1134	0.9157	1.4281	0.2366	0.5981	0.1147	2254	201	0.6264
A3F	1.0136	0.1944	0.6925	1.4835	0.9448	0.8526	0.0092	2254	201	0.9469
A3L	0.8819	0.0706	0.7680	1.0128	0.0752	0.0000	0.4713	2254	201	0.6264
A3L0F	0.9134	0.1520	0.6780	1.2304	0.5511	0.7491	0.0459	2254	201	0.7516
A3LF	1.0219	0.1757	0.7241	1.4420	0.9020	0.8229	0.0175	2254	201	0.9469
A3S	0.9628	0.0706	0.8384	1.1057	0.5911	0.0000	0.4181	2254	201	0.7823
A4E	1.1864	0.0947	0.9855	1.4283	0.0710	0.4400	0.1814	2254	201	0.6264
A4F	0.9860	0.2121	0.6507	1.4941	0.9469	0.8765	0.0044	2254	201	0.9469
A4F0GE	1.1717	0.0705	1.0206	1.3453	0.0245	0.0000	0.3555	2254	201	0.3677
A4F0GL	0.8511	0.0714	0.7400	0.9789	0.0239	0.0000	0.9140	2254	201	0.3677
A4FGE	1.0869	0.1025	0.8890	1.3288	0.4164	0.5125	0.1521	2254	201	0.7179
A4FGL	0.8905	0.0714	0.7742	1.0244	0.1046	0.0000	0.3594	2254	201	0.6264
A4FGS	0.9059	0.0718	0.7870	1.0429	0.1690	0.0000	0.8752	2254	201	0.6264
A4L	0.8254	0.0721	0.7166	0.9508	0.0078	0.0000	0.4179	2254	201	0.3526
A4S	0.8924	0.0723	0.7744	1.0283	0.1155	0.0000	0.4271	2254	201	0.6264
CA2	0.9842	0.0921	0.8217	1.1789	0.8631	0.3686	0.2082	2254	201	0.9469
CA3	1.0414	0.0935	0.8670	1.2509	0.6643	0.3944	0.1988	2254	201	0.8079
MHy	0.9948	0.0742	0.8601	1.1505	0.9435	0.0000	0.4966	2254	201	0.9469
MM	1.1161	0.2346	0.7048	1.7676	0.6395	0.9066	0.0011	2254	201	0.8079
TA2FS0	0.8809	0.1065	0.7149	1.0854	0.2339	0.4520	0.1767	2254	201	0.6264

Supplementary Table 7. Meta analysed associations between derived glycan traits and cardiovascular disease in type 2 diabetes in the full model. Glycan values are centered and scaled. The *p*-values after FDR correction that are significant (≤0.05) are highlighted in green. Red represents a positive association and blue a negative association. Full model: age, sex, the interaction thereof, BMI, HDL, non-HDL, duration of diabetes, eGFR and HbA1c.

Prevalent (cross-sectional)										
**glycome**	**OR**	**SE**	**CIlower**	**CIupper**	**pval**	**I2**	**I2pval**	**n**	**nevent**	**pval.FDR**
A2E	1.0513	0.0437	0.9650	1.1454	0.2525	0.0000	0.7037	3187	916	0.5345
A2E0F	0.8603	0.0434	0.7901	0.9367	0.0005	0.0000	0.9526	3187	916	0.0059
A2EF	0.9240	0.0436	0.8483	1.0065	0.0700	0.0000	0.9937	3187	916	0.2566
A2F	0.9239	0.0434	0.8486	1.0058	0.0679	0.0000	0.8748	3187	916	0.2566
A2F0G	0.9663	0.0426	0.8889	1.0505	0.4213	0.0000	0.5009	3187	916	0.6997
A2F0GE	0.9939	0.0447	0.9105	1.0849	0.8911	0.0000	0.8753	3187	916	0.9548
A2F0GL	0.9886	0.0512	0.8941	1.0930	0.8223	0.2189	0.2579	3187	916	0.9251
A2F0GS	0.9791	0.0440	0.8982	1.0674	0.6318	0.0000	0.6116	3187	916	0.8123
A2F0S0B	1.0930	0.0591	0.9735	1.2272	0.1321	0.4288	0.1858	3187	916	0.3526
A2F0S0G	0.9666	0.0436	0.8874	1.0528	0.4354	0.0000	0.5780	3187	916	0.6997
A2F0SB	1.0009	0.0423	0.9212	1.0874	0.9835	0.0000	0.7371	3187	916	0.9835
A2FG	1.0076	0.0447	0.9231	1.0999	0.8652	0.0000	0.9940	3187	916	0.9496
A2FGE	1.0233	0.0448	0.9372	1.1173	0.6068	0.0000	0.5005	3187	916	0.8123
A2FGL	1.1103	0.0697	0.9686	1.2728	0.1332	0.5656	0.1292	3187	916	0.3526
A2FGS	1.0829	0.0446	0.9923	1.1818	0.0741	0.0000	0.8579	3187	916	0.2566
A2FS0B	1.3275	0.0463	1.2122	1.4537	0.0000	0.0000	0.8541	3187	916	0.0000
A2FS0G	0.9319	0.0493	0.8462	1.0264	0.1525	0.0000	0.7556	3187	916	0.3813
A2FSB	1.0196	0.0483	0.9276	1.1208	0.6875	0.1796	0.2696	3187	916	0.8594
A2FSG	1.0029	0.0435	0.9209	1.0922	0.9470	0.0000	0.4107	3187	916	0.9685
A2GS	1.0598	0.0439	0.9724	1.1551	0.1862	0.0000	0.7982	3187	916	0.4411
A2L	1.0520	0.0451	0.9630	1.1492	0.2613	0.0000	0.8491	3187	916	0.5345
A2L0F	0.9171	0.0435	0.8422	0.9988	0.0468	0.0000	0.8866	3187	916	0.2566
A2LF	1.0093	0.1210	0.7961	1.2795	0.9393	0.8562	0.0084	3187	916	0.9685
A2S0F	0.8853	0.0423	0.8149	0.9618	0.0040	0.0000	0.9962	3187	916	0.0297
A2SG	1.0510	0.0428	0.9664	1.1430	0.2453	0.0000	0.5649	3187	916	0.5345
A3E	1.0782	0.0472	0.9829	1.1827	0.1108	0.0000	0.7343	3187	916	0.3325
A3F	0.9400	0.1237	0.7377	1.1979	0.6171	0.8581	0.0079	3187	916	0.8123
A3L	0.9171	0.0451	0.8395	1.0018	0.0549	0.0000	0.3582	3187	916	0.2566
A3L0F	0.8778	0.1274	0.6837	1.1268	0.3063	0.8605	0.0074	3187	916	0.5566
A3LF	0.9561	0.1209	0.7543	1.2118	0.7103	0.8533	0.0090	3187	916	0.8638
A3S	0.9575	0.0425	0.8810	1.0408	0.3075	0.0000	0.5476	3187	916	0.5566
A4E	1.1803	0.0449	1.0809	1.2888	0.0002	0.0000	0.3946	3187	916	0.0033
A4F	0.9694	0.1190	0.7677	1.2242	0.7941	0.8471	0.0106	3187	916	0.9162
A4F0GE	1.1955	0.0443	1.0961	1.3040	0.0001	0.0000	0.8833	3187	916	0.0012
A4F0GL	0.8670	0.0449	0.7939	0.9469	0.0015	0.0000	0.3540	3187	916	0.0135
A4FGE	1.0386	0.1136	0.8313	1.2977	0.7385	0.8401	0.0124	3187	916	0.8746
A4FGL	0.9268	0.1048	0.7547	1.1382	0.4686	0.8080	0.0225	3187	916	0.7271
A4FGS	0.9231	0.0443	0.8464	1.0068	0.0708	0.0000	0.4855	3187	916	0.2566
A4L	0.8618	0.0534	0.7762	0.9569	0.0053	0.2456	0.2496	3187	916	0.0343
A4S	0.9705	0.0510	0.8782	1.0726	0.5576	0.2405	0.2512	3187	916	0.7853
CA2	0.9569	0.0449	0.8762	1.0449	0.3263	0.0000	0.4668	3187	916	0.5648
CA3	1.0471	0.0453	0.9582	1.1443	0.3092	0.0000	0.5185	3187	916	0.5566
MHy	1.0268	0.0453	0.9397	1.1221	0.5585	0.0000	0.3827	3187	916	0.7853
MM	0.9721	0.0429	0.8937	1.0573	0.5087	0.0000	0.7026	3187	916	0.7631
TA2FS0	0.9285	0.0439	0.8520	1.0120	0.0913	0.0000	0.7557	3187	916	0.2934

Supplementary Table 7. Meta analysed associations between derived glycan traits and cardiovascular disease in type 2 diabetes in the full model. Glycan values are centered and scaled. The *p*-values after FDR correction that are significant (≤0.05) are highlighted in green. Red represents a positive association and blue a negative association. Full model: age, sex, the interaction thereof, BMI, HDL, non-HDL, duration of diabetes, eGFR and HbA1c.

Incident (Cox)										
**glycome**	**HR**	**SE**	**CIlower**	**CIupper**	**pval**	**I2**	**I2pval**	**n**	**nevent**	**pval.FDR**
A2E	1.1846	0.1654	0.8567	1.6381	0.3055	0.7729	0.0359	2254	201	0.6043
A2E0F	0.9525	0.0851	0.8062	1.1253	0.5671	0.2543	0.2468	2254	201	0.8004
A2EF	0.9647	0.0731	0.8359	1.1132	0.6226	0.0000	0.7950	2254	201	0.8004
A2F	0.9298	0.0745	0.8034	1.0761	0.3288	0.0000	0.6407	2254	201	0.6043
A2F0G	1.1434	0.0795	0.9784	1.3362	0.0920	0.0000	0.4506	2254	201	0.5913
A2F0GE	1.0858	0.0762	0.9352	1.2608	0.2800	0.0068	0.3157	2254	201	0.6043
A2F0GL	0.9374	0.0754	0.8086	1.0866	0.3910	0.0000	0.8900	2254	201	0.6516
A2F0GS	1.0543	0.0992	0.8679	1.2806	0.5944	0.4184	0.1898	2254	201	0.8004
A2F0S0B	0.9229	0.0734	0.7992	1.0656	0.2741	0.0000	0.3292	2254	201	0.6043
A2F0S0G	1.1048	0.0734	0.9568	1.2757	0.1746	0.0000	0.4900	2254	201	0.6043
A2F0SB	0.9338	0.0861	0.7887	1.1056	0.4267	0.1935	0.2655	2254	201	0.6621
A2FG	1.0359	0.1234	0.8134	1.3194	0.7748	0.6054	0.1114	2254	201	0.8953
A2FGE	1.1391	0.1631	0.8275	1.5680	0.4246	0.7760	0.0346	2254	201	0.6621
A2FGL	1.0777	0.0743	0.9317	1.2465	0.3139	0.0000	0.9415	2254	201	0.6043
A2FGS	1.1811	0.1634	0.8575	1.6269	0.3082	0.7773	0.0341	2254	201	0.6043
A2FS0B	0.9776	0.1838	0.6819	1.4016	0.9020	0.8233	0.0173	2254	201	0.9225
A2FS0G	0.8965	0.0839	0.7605	1.0568	0.1930	0.0000	0.4744	2254	201	0.6043
A2FSB	0.9230	0.0745	0.7976	1.0683	0.2827	0.0000	0.3339	2254	201	0.6043
A2FSG	1.1357	0.1359	0.8701	1.4823	0.3491	0.6696	0.0819	2254	201	0.6043
A2GS	1.1741	0.1473	0.8798	1.5670	0.2757	0.7218	0.0580	2254	201	0.6043
A2L	0.9863	0.0759	0.8499	1.1445	0.8555	0.0000	0.7671	2254	201	0.8953
A2L0F	0.9253	0.0744	0.7997	1.0707	0.2972	0.0000	0.7070	2254	201	0.6043
A2LF	1.0313	0.0756	0.8893	1.1961	0.6834	0.0000	0.3699	2254	201	0.8398
A2S0F	0.9599	0.0726	0.8326	1.1066	0.5724	0.0000	0.3520	2254	201	0.8004
A2SG	1.1106	0.0805	0.9485	1.3004	0.1924	0.0000	0.3652	2254	201	0.6043
A3E	1.1767	0.1354	0.9025	1.5342	0.2294	0.6575	0.0875	2254	201	0.6043
A3F	0.9561	0.1696	0.6856	1.3331	0.7911	0.7997	0.0255	2254	201	0.8953
A3L	0.8620	0.0755	0.7435	0.9995	0.0492	0.0000	0.4711	2254	201	0.5536
A3L0F	0.8698	0.1410	0.6598	1.1468	0.3228	0.6914	0.0719	2254	201	0.6043
A3LF	0.9674	0.1523	0.7177	1.3039	0.8277	0.7559	0.0430	2254	201	0.8953
A3S	0.9587	0.0715	0.8332	1.1030	0.5551	0.0000	0.3851	2254	201	0.8004
A4E	1.2070	0.1038	0.9848	1.4792	0.0699	0.5053	0.1551	2254	201	0.5913
A4F	0.9268	0.1910	0.6374	1.3475	0.6905	0.8426	0.0117	2254	201	0.8398
A4F0GE	1.1675	0.0771	1.0037	1.3580	0.0446	0.1407	0.2807	2254	201	0.5536
A4F0GL	0.8517	0.0737	0.7371	0.9841	0.0294	0.0000	0.7723	2254	201	0.5536
A4FGE	1.1066	0.0983	0.9127	1.3417	0.3027	0.4309	0.1850	2254	201	0.6043
A4FGL	0.8805	0.0742	0.7613	1.0184	0.0864	0.0000	0.3376	2254	201	0.5913
A4FGS	0.9157	0.0734	0.7930	1.0575	0.2305	0.0000	0.7199	2254	201	0.6043
A4L	0.8043	0.0778	0.6906	0.9368	0.0051	0.0318	0.3095	2254	201	0.2305
A4S	0.8859	0.0754	0.7642	1.0270	0.1080	0.0000	0.4167	2254	201	0.6043
CA2	1.0031	0.1160	0.7990	1.2592	0.9790	0.5877	0.1194	2254	201	0.9790
CA3	1.0221	0.1156	0.8149	1.2820	0.8498	0.5893	0.1187	2254	201	0.8953
MHy	1.0150	0.0740	0.8780	1.1735	0.8403	0.0000	0.6956	2254	201	0.8953
MM	1.1236	0.2326	0.7122	1.7726	0.6163	0.9062	0.0011	2254	201	0.8004
TA2FS0	0.8888	0.1231	0.6983	1.1314	0.3384	0.5710	0.1268	2254	201	0.6043

Supplementary Table 8. Meta analysed associations between derived glycan traits and nephropathy in type 2 diabetes in the basic model. Glycan values are centered and scaled. The *p*-values after FDR correction that are significant (≤0.05) are highlighted in green. Red represents a positive association and blue a negative association. Basic model: age, sex and the interaction thereof.

Prevalent (cross-sectional)										
**glycome**	**OR**	**SE**	**CIlower**	**CIupper**	**pval**	**I2**	**I2pval**	**n**	**nevent**	**pval.FDR**
A2E	1.0984	0.0920	0.9171	1.3155	0.3077	0.7281	0.0552	3139	603	0.5273
A2E0F	0.9545	0.0467	0.8710	1.0460	0.3187	0.0000	0.9290	3139	603	0.5273
A2EF	0.9809	0.1089	0.7923	1.2144	0.8595	0.8159	0.0198	3139	603	0.9540
A2F	0.9580	0.0967	0.7925	1.1580	0.6572	0.7617	0.0405	3139	603	0.8215
A2F0G	1.0007	0.0580	0.8931	1.1212	0.9910	0.3483	0.2154	3139	603	0.9910
A2F0GE	1.0682	0.0952	0.8864	1.2873	0.4884	0.7394	0.0501	3139	603	0.7089
A2F0GL	0.8551	0.0487	0.7772	0.9408	0.0013	0.0000	0.5169	3139	603	0.0084
A2F0GS	1.0132	0.0796	0.8669	1.1842	0.8692	0.6293	0.1005	3139	603	0.9540
A2F0S0B	1.1525	0.0474	1.0503	1.2648	0.0027	0.0000	0.6801	3139	603	0.0137
A2F0S0G	0.8981	0.0472	0.8187	0.9852	0.0228	0.0000	0.7570	3139	603	0.0642
A2F0SB	1.0044	0.0500	0.9106	1.1078	0.9303	0.1419	0.2804	3139	603	0.9702
A2FG	0.9566	0.0481	0.8705	1.0513	0.3567	0.0000	0.6842	3139	603	0.5536
A2FGE	1.1818	0.0473	1.0771	1.2967	0.0004	0.0000	0.4080	3139	603	0.0038
A2FGL	1.0409	0.0663	0.9141	1.1853	0.5451	0.4794	0.1657	3139	603	0.7665
A2FGS	1.2139	0.0482	1.1044	1.3343	0.0001	0.0000	0.8468	3139	603	0.0012
A2FS0B	1.1772	0.0482	1.0711	1.2939	0.0007	0.0000	0.6833	3139	603	0.0054
A2FS0G	0.8219	0.0525	0.7415	0.9109	0.0002	0.0000	0.9775	3139	603	0.0021
A2FSB	1.0222	0.1240	0.8017	1.3035	0.8593	0.8507	0.0096	3139	603	0.9540
A2FSG	1.0112	0.0478	0.9207	1.1106	0.8159	0.0000	0.5781	3139	603	0.9540
A2GS	1.1174	0.0921	0.9328	1.3384	0.2283	0.7230	0.0574	3139	603	0.4466
A2L	0.8838	0.0482	0.8041	0.9714	0.0104	0.0000	0.7885	3139	603	0.0389
A2L0F	0.9555	0.0973	0.7897	1.1562	0.6398	0.7638	0.0396	3139	603	0.8215
A2LF	1.0633	0.0531	0.9581	1.1800	0.2484	0.2003	0.2635	3139	603	0.4471
A2S0F	0.9415	0.0457	0.8608	1.0298	0.1874	0.0000	0.7008	3139	603	0.3833
A2SG	1.0074	0.0761	0.8678	1.1694	0.9231	0.6433	0.0941	3139	603	0.9702
A3E	1.2795	0.0475	1.1657	1.4044	0.0000	0.0000	0.4733	3139	603	0.0000
A3F	1.0089	0.0508	0.9132	1.1147	0.8609	0.0996	0.2920	3139	603	0.9540
A3L	0.8649	0.0466	0.7894	0.9476	0.0018	0.0000	0.4079	3139	603	0.0104
A3L0F	0.9383	0.0481	0.8539	1.0311	0.1858	0.0000	0.6205	3139	603	0.3833
A3LF	1.0272	0.0540	0.9241	1.1418	0.6191	0.2014	0.2631	3139	603	0.8193
A3S	1.0564	0.0470	0.9634	1.1584	0.2435	0.0000	0.8136	3139	603	0.4471
A4E	1.0888	0.0870	0.9181	1.2911	0.3281	0.7004	0.0677	3139	603	0.5273
A4F	1.0031	0.0483	0.9125	1.1028	0.9486	0.0000	0.4609	3139	603	0.9702
A4F0GE	1.0592	0.1052	0.8618	1.3018	0.5845	0.7976	0.0262	3139	603	0.7970
A4F0GL	0.8942	0.0665	0.7850	1.0186	0.0924	0.4985	0.1579	3139	603	0.2310
A4FGE	1.1188	0.0462	1.0218	1.2249	0.0152	0.0000	0.8624	3139	603	0.0457
A4FGL	0.8748	0.0462	0.7991	0.9576	0.0038	0.0000	0.3539	3139	603	0.0154
A4FGS	0.9117	0.0618	0.8077	1.0292	0.1349	0.4106	0.1927	3139	603	0.3196
A4L	0.8767	0.0756	0.7559	1.0168	0.0819	0.6049	0.1116	3139	603	0.2167
A4S	0.8715	0.0471	0.7946	0.9558	0.0035	0.0000	0.6334	3139	603	0.0154
CA2	0.8884	0.0481	0.8084	0.9763	0.0140	0.0000	0.4361	3139	603	0.0457
CA3	1.1245	0.0483	1.0229	1.2363	0.0152	0.0000	0.3676	3139	603	0.0457
MHy	0.8168	0.0513	0.7387	0.9032	0.0001	0.0000	0.8161	3139	603	0.0012
MM	1.0333	0.0469	0.9426	1.1328	0.4844	0.0000	0.7396	3139	603	0.7089
TA2FS0	0.9300	0.0501	0.8430	1.0261	0.1481	0.0469	0.3057	3139	603	0.3332

Supplementary Table 8. Meta analysed associations between derived glycan traits and nephropathy in type 2 diabetes in the basic model. Glycan values are centered and scaled. The *p*-values after FDR correction that are significant (≤0.05) are highlighted in green. Red represents a positive association and blue a negative association. Basic model: age, sex and the interaction thereof.

	Incident (Cox)									
**glycome**	**HR**	**SE**	**CIlower**	**CIupper**	**pval**	**I2**	**I2pval**	**n**	**nevent**	**pval.FDR**
A2E	1.0488	0.0517	0.9477	1.1607	0.3566	0.0000	0.8910	2486	402	0.7294
A2E0F	0.9853	0.0950	0.8180	1.1868	0.8760	0.6900	0.0725	2486	402	0.9615
A2EF	1.0198	0.0623	0.9025	1.1522	0.7533	0.3041	0.2306	2486	402	0.9615
A2F	0.9824	0.0538	0.8841	1.0917	0.7418	0.0559	0.3034	2486	402	0.9615
A2F0G	0.9869	0.0511	0.8929	1.0909	0.7965	0.0000	0.4018	2486	402	0.9615
A2F0GE	0.9858	0.0512	0.8917	1.0899	0.7800	0.0000	0.6138	2486	402	0.9615
A2F0GL	0.9382	0.0516	0.8479	1.0380	0.2159	0.0000	0.9423	2486	402	0.6478
A2F0GS	0.9437	0.0499	0.8559	1.0407	0.2456	0.0000	0.3492	2486	402	0.6502
A2F0S0B	1.1745	0.0923	0.9801	1.4074	0.0815	0.6799	0.0772	2486	402	0.2821
A2F0S0G	0.8778	0.0696	0.7659	1.0060	0.0610	0.4341	0.1837	2486	402	0.2286
A2F0SB	1.0020	0.0509	0.9068	1.1071	0.9692	0.0000	0.7914	2486	402	0.9978
A2FG	0.9574	0.0525	0.8637	1.0612	0.4068	0.0000	0.4419	2486	402	0.7959
A2FGE	1.1533	0.0495	1.0467	1.2707	0.0040	0.0000	0.7485	2486	402	0.0593
A2FGL	0.9985	0.0639	0.8810	1.1318	0.9814	0.3241	0.2239	2486	402	0.9978
A2FGS	1.1692	0.0508	1.0584	1.2915	0.0021	0.0000	0.7823	2486	402	0.0469
A2FS0B	1.1225	0.0611	0.9959	1.2652	0.0584	0.2527	0.2474	2486	402	0.2286
A2FS0G	0.8115	0.0569	0.7258	0.9073	0.0002	0.0000	0.3501	2486	402	0.0110
A2FSB	1.0565	0.0516	0.9548	1.1690	0.2870	0.0000	0.6031	2486	402	0.6798
A2FSG	1.0287	0.0819	0.8761	1.2079	0.7294	0.5684	0.1280	2486	402	0.9615
A2GS	1.0543	0.0516	0.9529	1.1665	0.3058	0.0000	0.7028	2486	402	0.6880
A2L	0.9467	0.0513	0.8561	1.0468	0.2854	0.0000	0.9033	2486	402	0.6798
A2L0F	0.9860	0.0521	0.8903	1.0920	0.7865	0.0000	0.3234	2486	402	0.9615
A2LF	0.9964	0.0516	0.9006	1.1024	0.9445	0.0000	0.4485	2486	402	0.9978
A2S0F	1.0164	0.0693	0.8874	1.1642	0.8138	0.4539	0.1760	2486	402	0.9615
A2SG	0.9850	0.0537	0.8867	1.0943	0.7785	0.0000	0.5456	2486	402	0.9615
A3E	1.0979	0.0605	0.9752	1.2361	0.1226	0.2751	0.2402	2486	402	0.3939
A3F	0.9653	0.0529	0.8703	1.0708	0.5046	0.0000	0.6565	2486	402	0.8734
A3L	0.8659	0.0633	0.7649	0.9802	0.0228	0.3449	0.2166	2486	402	0.1462
A3L0F	0.9515	0.0529	0.8577	1.0556	0.3481	0.0000	0.3605	2486	402	0.7294
A3LF	0.9839	0.0526	0.8875	1.0907	0.7571	0.0000	0.7801	2486	402	0.9615
A3S	0.9096	0.0490	0.8264	1.0013	0.0531	0.0000	0.7406	2486	402	0.2286
A4E	1.1152	0.0505	1.0101	1.2312	0.0308	0.0000	0.7563	2486	402	0.1541
A4F	0.9629	0.0529	0.8681	1.0681	0.4748	0.0000	0.3849	2486	402	0.8546
A4F0GE	1.1352	0.0503	1.0286	1.2528	0.0117	0.0000	0.4760	2486	402	0.0891
A4F0GL	0.8798	0.0509	0.7962	0.9721	0.0119	0.0000	0.4399	2486	402	0.0891
A4FGE	1.0522	0.0679	0.9211	1.2018	0.4537	0.4261	0.1868	2486	402	0.8507
A4FGL	0.9792	0.0514	0.8853	1.0829	0.6820	0.0000	0.7045	2486	402	0.9615
A4FGS	1.0094	0.0535	0.9090	1.1209	0.8613	0.0422	0.3069	2486	402	0.9615
A4L	0.8909	0.0519	0.8047	0.9863	0.0260	0.0127	0.3142	2486	402	0.1462
A4S	0.9462	0.0884	0.7956	1.1253	0.5316	0.6548	0.0888	2486	402	0.8860
CA2	0.9866	0.0760	0.8500	1.1451	0.8588	0.5074	0.1542	2486	402	0.9615
CA3	1.0002	0.0803	0.8546	1.1707	0.9978	0.5571	0.1329	2486	402	0.9978
MHy	0.8686	0.0547	0.7803	0.9669	0.0100	0.0000	0.6262	2486	402	0.0891
MM	1.0218	0.0495	0.9273	1.1259	0.6630	0.0000	0.9692	2486	402	0.9615
TA2FS0	0.9379	0.0541	0.8436	1.0427	0.2357	0.0000	0.4758	2486	402	0.6502

Supplementary Table 9. Meta analysed associations between derived glycan traits and nephropathy in type 2 diabetes in the full model. Glycan values are centered and scaled. The *p*-values after FDR correction that are significant (≤0.05) are highlighted in green. Red represents a positive association and blue a negative association. Full model: age, sex, the interaction thereof, BMI, HDL, non-HDL, duration of diabetes, eGFR and HbA1c.

Prevalent (cross-sectional)										
**glycome**	**OR**	**SE**	**CIlower**	**CIupper**	**pval**	**I2**	**I2pval**	**n**	**nevent**	**pval.FDR**
A2E	1.0234	0.1063	0.8309	1.2605	0.8280	0.7749	0.0351	3139	603	0.9819
A2E0F	1.0044	0.0492	0.9120	1.1060	0.9294	0.0000	0.8278	3139	603	0.9819
A2EF	0.9957	0.1193	0.7881	1.2580	0.9712	0.8318	0.0148	3139	603	0.9819
A2F	0.9959	0.1105	0.8019	1.2369	0.9706	0.8007	0.0251	3139	603	0.9819
A2F0G	0.9801	0.0596	0.8721	1.1015	0.7355	0.3454	0.2165	3139	603	0.9819
A2F0GE	1.0312	0.1051	0.8393	1.2671	0.7699	0.7571	0.0425	3139	603	0.9819
A2F0GL	0.9134	0.0554	0.8195	1.0182	0.1022	0.1345	0.2824	3139	603	0.4601
A2F0GS	1.0043	0.0841	0.8516	1.1843	0.9594	0.6257	0.1022	3139	603	0.9819
A2F0S0B	1.1213	0.0493	1.0180	1.2351	0.0202	0.0000	0.7033	3139	603	0.2276
A2F0S0G	0.9191	0.0494	0.8342	1.0125	0.0876	0.0000	0.4074	3139	603	0.4601
A2F0SB	1.0118	0.0478	0.9214	1.1112	0.8056	0.0000	0.3335	3139	603	0.9819
A2FG	0.9332	0.0500	0.8460	1.0293	0.1670	0.0000	0.7904	3139	603	0.6612
A2FGE	1.0960	0.0507	0.9923	1.2106	0.0707	0.0000	0.3192	3139	603	0.4601
A2FGL	1.0068	0.0926	0.8396	1.2072	0.9418	0.6784	0.0778	3139	603	0.9819
A2FGS	1.1078	0.0510	1.0025	1.2242	0.0445	0.0000	0.8582	3139	603	0.4008
A2FS0B	1.0725	0.0518	0.9690	1.1871	0.1763	0.0000	0.8748	3139	603	0.6612
A2FS0G	0.8692	0.0552	0.7800	0.9686	0.0112	0.0000	0.9889	3139	603	0.1676
A2FSB	1.0437	0.1204	0.8243	1.3216	0.7224	0.8307	0.0151	3139	603	0.9819
A2FSG	0.9769	0.0497	0.8862	1.0768	0.6378	0.0000	0.8390	3139	603	0.9819
A2GS	1.0486	0.1002	0.8616	1.2761	0.6358	0.7388	0.0504	3139	603	0.9819
A2L	0.9164	0.0520	0.8277	1.0146	0.0928	0.0000	0.6536	3139	603	0.4601
A2L0F	0.9974	0.1135	0.7985	1.2459	0.9819	0.8093	0.0220	3139	603	0.9819
A2LF	1.0118	0.0501	0.9173	1.1162	0.8141	0.0000	0.5899	3139	603	0.9819
A2S0F	0.9774	0.0482	0.8893	1.0744	0.6363	0.0000	0.4474	3139	603	0.9819
A2SG	0.9705	0.0847	0.8221	1.1457	0.7238	0.6937	0.0708	3139	603	0.9819
A3E	1.1677	0.0527	1.0532	1.2947	0.0032	0.0000	0.6268	3139	603	0.1459
A3F	0.9678	0.0510	0.8757	1.0697	0.5216	0.0000	0.3814	3139	603	0.9819
A3L	0.9571	0.0505	0.8669	1.0568	0.3856	0.0000	0.3289	3139	603	0.9819
A3L0F	0.9472	0.0505	0.8579	1.0458	0.2828	0.0000	0.7073	3139	603	0.9089
A3LF	0.9704	0.0509	0.8782	1.0722	0.5547	0.0000	0.3798	3139	603	0.9819
A3S	1.0864	0.0492	0.9866	1.1964	0.0919	0.0000	0.4672	3139	603	0.4601
A4E	1.0080	0.0963	0.8346	1.2173	0.9343	0.7168	0.0602	3139	603	0.9819
A4F	0.9600	0.0513	0.8683	1.0615	0.4261	0.0000	0.5787	3139	603	0.9819
A4F0GE	0.9801	0.1065	0.7954	1.2076	0.8500	0.7740	0.0354	3139	603	0.9819
A4F0GL	1.0023	0.0828	0.8521	1.1789	0.9782	0.6202	0.1047	3139	603	0.9819
A4FGE	1.0549	0.0494	0.9576	1.1622	0.2791	0.0000	0.4470	3139	603	0.9089
A4FGL	0.9496	0.0720	0.8247	1.0935	0.4728	0.5100	0.1531	3139	603	0.9819
A4FGS	0.9834	0.0802	0.8403	1.1508	0.8344	0.6032	0.1124	3139	603	0.9819
A4L	0.9745	0.0883	0.8196	1.1586	0.7697	0.6564	0.0880	3139	603	0.9819
A4S	0.9543	0.0497	0.8657	1.0520	0.3472	0.0000	0.6704	3139	603	0.9819
CA2	0.9336	0.0784	0.8006	1.0887	0.3807	0.5724	0.1262	3139	603	0.9819
CA3	1.0625	0.0792	0.9097	1.2410	0.4440	0.5758	0.1247	3139	603	0.9819
MHy	0.8679	0.0534	0.7817	0.9636	0.0079	0.0000	0.4378	3139	603	0.1676
MM	1.0297	0.0490	0.9354	1.1335	0.5504	0.0000	0.3604	3139	603	0.9819
TA2FS0	0.9947	0.0578	0.8883	1.1140	0.9271	0.2309	0.2542	3139	603	0.9819

Supplementary Table 9. Meta analysed associations between derived glycan traits and nephropathy in type 2 diabetes in the full model. Glycan values are centered and scaled. The *p*-values after FDR correction that are significant (≤0.05) are highlighted in green. Red represents a positive association and blue a negative association. Full model: age, sex, the interaction thereof, BMI, HDL, non-HDL, duration of diabetes, eGFR and HbA1c.

Incident (Cox)										
**glycome**	**HR**	**SE**	**CIlower**	**CIupper**	**pval**	**I2**	**I2pval**	**n**	**nevent**	**pval.FDR**
A2E	0.9973	0.0521	0.9005	1.1044	0.9580	0.0000	0.9264	2486	402	0.9946
A2E0F	0.9746	0.0823	0.8294	1.1451	0.7541	0.5768	0.1242	2486	402	0.9946
A2EF	1.0087	0.0533	0.9087	1.1196	0.8713	0.0000	0.4414	2486	402	0.9946
A2F	0.9833	0.0531	0.8862	1.0912	0.7515	0.0000	0.4795	2486	402	0.9946
A2F0G	0.9712	0.0553	0.8715	1.0823	0.5969	0.1213	0.2861	2486	402	0.9946
A2F0GE	0.9200	0.0526	0.8299	1.0198	0.1126	0.0000	0.6441	2486	402	0.7424
A2F0GL	0.9994	0.0532	0.9005	1.1092	0.9912	0.0000	0.8378	2486	402	0.9946
A2F0GS	0.8999	0.0507	0.8148	0.9940	0.0376	0.0000	0.3851	2486	402	0.4228
A2F0S0B	1.1348	0.0910	0.9494	1.3564	0.1647	0.6638	0.0846	2486	402	0.7424
A2F0S0G	0.9122	0.0619	0.8079	1.0299	0.1377	0.2715	0.2413	2486	402	0.7424
A2F0SB	1.0142	0.0517	0.9165	1.1224	0.7851	0.0000	0.5708	2486	402	0.9946
A2FG	0.9650	0.0526	0.8704	1.0697	0.4977	0.0000	0.5860	2486	402	0.9946
A2FGE	1.0848	0.0519	0.9798	1.2011	0.1170	0.0000	0.7845	2486	402	0.7424
A2FGL	1.0290	0.0550	0.9239	1.1460	0.6029	0.0244	0.3113	2486	402	0.9946
A2FGS	1.1083	0.0520	1.0009	1.2273	0.0480	0.0000	0.7576	2486	402	0.4324
A2FS0B	1.0795	0.0551	0.9690	1.2026	0.1650	0.0328	0.3092	2486	402	0.7424
A2FS0G	0.8588	0.0582	0.7663	0.9625	0.0088	0.0000	0.4380	2486	402	0.3979
A2FSB	1.0684	0.0522	0.9644	1.1835	0.2053	0.0000	0.4410	2486	402	0.8398
A2FSG	1.0073	0.0673	0.8827	1.1494	0.9140	0.3662	0.2091	2486	402	0.9946
A2GS	0.9996	0.0523	0.9023	1.1075	0.9946	0.0000	0.7212	2486	402	0.9946
A2L	1.0071	0.0536	0.9067	1.1187	0.8944	0.0000	0.9561	2486	402	0.9946
A2L0F	0.9864	0.0532	0.8888	1.0948	0.7968	0.0000	0.4960	2486	402	0.9946
A2LF	0.9767	0.0533	0.8798	1.0842	0.6577	0.0000	0.7150	2486	402	0.9946
A2S0F	1.0074	0.0607	0.8945	1.1346	0.9026	0.2877	0.2361	2486	402	0.9946
A2SG	0.9693	0.0540	0.8720	1.0775	0.5637	0.0000	0.3373	2486	402	0.9946
A3E	0.9610	0.0564	0.8605	1.0733	0.4804	0.0000	0.4319	2486	402	0.9946
A3F	0.9746	0.0544	0.8761	1.0843	0.6368	0.0000	0.7632	2486	402	0.9946
A3L	0.9448	0.0698	0.8240	1.0833	0.4157	0.3894	0.2006	2486	402	0.9946
A3L0F	0.9970	0.0549	0.8953	1.1102	0.9560	0.0000	0.4844	2486	402	0.9946
A3LF	0.9803	0.0539	0.8821	1.0895	0.7121	0.0000	0.9131	2486	402	0.9946
A3S	0.8986	0.0496	0.8154	0.9904	0.0312	0.0000	0.4249	2486	402	0.4228
A4E	1.0334	0.0527	0.9319	1.1459	0.5334	0.0000	0.7256	2486	402	0.9946
A4F	0.9751	0.0542	0.8767	1.0845	0.6418	0.0000	0.4491	2486	402	0.9946
A4F0GE	1.0607	0.0521	0.9577	1.1749	0.2582	0.0000	0.4859	2486	402	0.8938
A4F0GL	0.9606	0.0536	0.8647	1.0671	0.4534	0.0000	0.3822	2486	402	0.9946
A4FGE	0.9809	0.0634	0.8663	1.1107	0.7611	0.2895	0.2355	2486	402	0.9946
A4FGL	1.0648	0.0545	0.9569	1.1848	0.2495	0.0000	0.8624	2486	402	0.8938
A4FGS	1.0727	0.0798	0.9174	1.2544	0.3792	0.5296	0.1448	2486	402	0.9946
A4L	0.9854	0.0613	0.8739	1.1113	0.8109	0.1904	0.2664	2486	402	0.9946
A4S	1.0171	0.0876	0.8566	1.2076	0.8469	0.6233	0.1032	2486	402	0.9946
CA2	1.0414	0.0542	0.9365	1.1581	0.4539	0.0000	0.3631	2486	402	0.9946
CA3	0.9405	0.0589	0.8380	1.0556	0.2979	0.1414	0.2805	2486	402	0.9574
MHy	0.8779	0.0554	0.7877	0.9786	0.0187	0.0000	0.4687	2486	402	0.4208
MM	1.0043	0.0504	0.9099	1.1086	0.9314	0.0000	0.7145	2486	402	0.9946
TA2FS0	0.9585	0.0540	0.8622	1.0656	0.4326	0.0000	0.5959	2486	402	0.9946

Supplementary Table 10. Meta analysed associations between derived glycan traits and retinopathy in type 2 diabetes in the basic model. Glycan values are centered and scaled. The *p*-values after FDR correction that are significant (≤0.05) are highlighted in green. Red represents a positive association and blue a negative association. Basic model: age, sex and the interaction thereof.

Prevalent (cross-sectional)										
**glycome**	**OR**	**SE**	**CIlower**	**CIupper**	**pval**	**I2**	**I2pval**	**n**	**nevent**	**pval.FDR**
A2E	0.9297	0.0479	0.8464	1.0212	0.1281	0.0000	0.7989	3217	505	0.4794
A2E0F	0.9484	0.0491	0.8614	1.0441	0.2799	0.0000	0.7490	3217	505	0.5432
A2EF	1.0741	0.0597	0.9555	1.2074	0.2310	0.3359	0.2198	3217	505	0.5198
A2F	1.0658	0.0481	0.9699	1.1712	0.1851	0.0000	0.4503	3217	505	0.4794
A2F0G	0.9210	0.0818	0.7846	1.0812	0.3145	0.6525	0.0898	3217	505	0.5662
A2F0GE	0.8682	0.0492	0.7884	0.9561	0.0041	0.0000	0.3525	3217	505	0.0917
A2F0GL	1.0423	0.0492	0.9466	1.1478	0.3990	0.0000	0.7585	3217	505	0.6905
A2F0GS	0.8585	0.0652	0.7555	0.9756	0.0194	0.4344	0.1836	3217	505	0.2903
A2F0S0B	0.9943	0.0500	0.9015	1.0967	0.9092	0.0000	0.5959	3217	505	0.9742
A2F0S0G	1.0873	0.0503	0.9852	1.2000	0.0962	0.0000	0.8250	3217	505	0.4558
A2F0SB	1.0809	0.0552	0.9702	1.2043	0.1583	0.2517	0.2477	3217	505	0.4794
A2FG	1.0112	0.0509	0.9153	1.1172	0.8260	0.0000	0.3632	3217	505	0.9530
A2FGE	1.0841	0.0497	0.9835	1.1949	0.1041	0.0000	0.4697	3217	505	0.4558
A2FGL	0.9997	0.0499	0.9065	1.1025	0.9949	0.0000	0.7366	3217	505	0.9997
A2FGS	1.0852	0.0499	0.9840	1.1968	0.1017	0.0000	0.6134	3217	505	0.4558
A2FS0B	1.0275	0.0509	0.9299	1.1352	0.5943	0.0000	0.9443	3217	505	0.9209
A2FS0G	0.9424	0.0553	0.8456	1.0503	0.2834	0.0000	0.6726	3217	505	0.5432
A2FSB	1.0059	0.0497	0.9126	1.1088	0.9056	0.0000	0.7342	3217	505	0.9742
A2FSG	1.0233	0.0501	0.9276	1.1288	0.6458	0.0000	0.8693	3217	505	0.9209
A2GS	0.9409	0.0481	0.8562	1.0339	0.2053	0.0000	0.6727	3217	505	0.4862
A2L	1.0667	0.0488	0.9693	1.1738	0.1862	0.0000	0.7050	3217	505	0.4794
A2L0F	1.0558	0.0482	0.9607	1.1604	0.2597	0.0000	0.4882	3217	505	0.5432
A2LF	1.0884	0.0514	0.9841	1.2037	0.0992	0.0469	0.3057	3217	505	0.4558
A2S0F	0.9722	0.0486	0.8839	1.0693	0.5612	0.0000	0.9536	3217	505	0.9020
A2SG	0.9250	0.0737	0.8006	1.0687	0.2897	0.5825	0.1217	3217	505	0.5432
A3E	0.9176	0.0492	0.8332	1.0106	0.0807	0.0000	0.3895	3217	505	0.4558
A3F	1.1044	0.0624	0.9773	1.2479	0.1114	0.3299	0.2219	3217	505	0.4558
A3L	0.9880	0.0492	0.8971	1.0880	0.8058	0.0000	0.5659	3217	505	0.9530
A3L0F	1.1379	0.0958	0.9430	1.3730	0.1778	0.7245	0.0567	3217	505	0.4794
A3LF	1.0969	0.0626	0.9703	1.2402	0.1394	0.3388	0.2188	3217	505	0.4794
A3S	0.8977	0.0827	0.7633	1.0556	0.1917	0.6474	0.0922	3217	505	0.4794
A4E	1.0167	0.0493	0.9230	1.1198	0.7374	0.0000	0.9991	3217	505	0.9217
A4F	1.1197	0.0507	1.0137	1.2367	0.0259	0.0000	0.3631	3217	505	0.2910
A4F0GE	1.0347	0.0490	0.9399	1.1390	0.4868	0.0000	0.3900	3217	505	0.8114
A4F0GL	0.9787	0.0489	0.8893	1.0770	0.6592	0.0000	0.3838	3217	505	0.9209
A4FGE	0.9840	0.0678	0.8617	1.1238	0.8121	0.4642	0.1719	3217	505	0.9530
A4FGL	1.0240	0.0567	0.9163	1.1444	0.6753	0.2408	0.2511	3217	505	0.9209
A4FGS	0.9998	0.0498	0.9069	1.1023	0.9972	0.0000	0.9860	3217	505	0.9997
A4L	1.0000	0.0492	0.9082	1.1012	0.9997	0.0000	0.8976	3217	505	0.9997
A4S	1.0183	0.0493	0.9246	1.1216	0.7121	0.0000	0.8831	3217	505	0.9217
CA2	1.0231	0.0512	0.9253	1.1312	0.6560	0.0000	0.3275	3217	505	0.9209
CA3	0.9757	0.0707	0.8495	1.1208	0.7281	0.4675	0.1706	3217	505	0.9217
MHy	0.7902	0.0718	0.6865	0.9096	0.0010	0.4033	0.1955	3217	505	0.0468
MM	0.9049	0.0488	0.8223	0.9958	0.0407	0.0000	0.6591	3217	505	0.3663
TA2FS0	1.0073	0.0482	0.9165	1.1070	0.8802	0.0000	0.9420	3217	505	0.9742

Supplementary Table 10. Meta analysed associations between derived glycan traits and retinopathy in type 2 diabetes in the basic model. Glycan values are centered and scaled. The *p*-values after FDR correction that are significant (≤0.05) are highlighted in green. Red represents a positive association and blue a negative association. Basic model: age, sex and the interaction thereof.

Incident (Cox)										
**glycome**	**HR**	**SE**	**CIlower**	**CIupper**	**pval**	**I2**	**I2pval**	**n**	**nevent**	**pval.FDR**
A2E	0.9376	0.0550	0.8417	1.0444	0.2416	0.0000	0.8196	2639	345	0.3884
A2E0F	1.0242	0.0928	0.8538	1.2285	0.7971	0.6017	0.1131	2639	345	0.8540
A2EF	1.0841	0.0801	0.9267	1.2683	0.3131	0.5566	0.1332	2639	345	0.4403
A2F	1.1029	0.0642	0.9724	1.2509	0.1273	0.2668	0.2429	2639	345	0.3370
A2F0G	0.8960	0.0867	0.7560	1.0619	0.2053	0.5888	0.1189	2639	345	0.3851
A2F0GE	0.9898	0.0563	0.8864	1.1051	0.8547	0.0448	0.3062	2639	345	0.8741
A2F0GL	0.9557	0.0559	0.8565	1.0665	0.4181	0.0000	0.7643	2639	345	0.5534
A2F0GS	0.9710	0.0749	0.8384	1.1246	0.6941	0.4443	0.1798	2639	345	0.8220
A2F0S0B	1.1013	0.0549	0.9890	1.2265	0.0787	0.0000	0.8362	2639	345	0.3370
A2F0S0G	0.8816	0.0539	0.7931	0.9799	0.0195	0.0000	0.6203	2639	345	0.2922
A2F0SB	1.1576	0.0522	1.0451	1.2823	0.0050	0.0000	0.3550	2639	345	0.2266
A2FG	0.9397	0.0561	0.8418	1.0490	0.2677	0.0000	0.6005	2639	345	0.4153
A2FGE	1.0883	0.1004	0.8939	1.3249	0.3995	0.6830	0.0757	2639	345	0.5448
A2FGL	0.9822	0.0661	0.8629	1.1181	0.7864	0.2487	0.2486	2639	345	0.8540
A2FGS	1.0867	0.0690	0.9493	1.2440	0.2279	0.3212	0.2248	2639	345	0.3851
A2FS0B	1.0574	0.0550	0.9493	1.1779	0.3104	0.0000	0.8565	2639	345	0.4403
A2FS0G	0.8660	0.1202	0.6843	1.0959	0.2310	0.7138	0.0616	2639	345	0.3851
A2FSB	1.0236	0.0551	0.9188	1.1402	0.6723	0.0000	0.9519	2639	345	0.8177
A2FSG	0.9882	0.0559	0.8857	1.1026	0.8319	0.0000	0.9058	2639	345	0.8706
A2GS	0.9748	0.0549	0.8754	1.0856	0.6425	0.0000	0.3615	2639	345	0.8032
A2L	0.9597	0.0560	0.8599	1.0710	0.4620	0.0000	0.9809	2639	345	0.5940
A2L0F	1.0964	0.0673	0.9610	1.2510	0.1713	0.3368	0.2195	2639	345	0.3846
A2LF	1.1033	0.0575	0.9857	1.2351	0.0874	0.0000	0.9693	2639	345	0.3370
A2S0F	1.0064	0.0899	0.8438	1.2003	0.9438	0.5930	0.1170	2639	345	0.9438
A2SG	0.9035	0.0503	0.8187	0.9971	0.0437	0.0000	0.9930	2639	345	0.3370
A3E	1.0735	0.0539	0.9659	1.1930	0.1880	0.0000	0.3649	2639	345	0.3846
A3F	1.0921	0.0572	0.9763	1.2217	0.1235	0.0000	0.3482	2639	345	0.3370
A3L	0.9014	0.0545	0.8102	1.0030	0.0568	0.0000	0.9659	2639	345	0.3370
A3L0F	1.0864	0.0571	0.9713	1.2152	0.1468	0.0000	0.8776	2639	345	0.3670
A3LF	1.1036	0.0589	0.9834	1.2386	0.0939	0.0650	0.3011	2639	345	0.3370
A3S	0.9187	0.0674	0.8051	1.0484	0.2083	0.3557	0.2128	2639	345	0.3851
A4E	1.1064	0.0547	0.9939	1.2316	0.0647	0.0000	0.8169	2639	345	0.3370
A4F	1.0973	0.0609	0.9739	1.2363	0.1273	0.0985	0.2923	2639	345	0.3370
A4F0GE	1.1030	0.0549	0.9905	1.2283	0.0740	0.0000	0.7876	2639	345	0.3370
A4F0GL	0.9142	0.0548	0.8210	1.0179	0.1018	0.0000	0.9293	2639	345	0.3370
A4FGE	1.0867	0.0533	0.9789	1.2064	0.1187	0.0000	0.8300	2639	345	0.3370
A4FGL	0.9293	0.0547	0.8347	1.0345	0.1800	0.0000	0.6561	2639	345	0.3846
A4FGS	0.9747	0.0792	0.8346	1.1383	0.7460	0.4773	0.1666	2639	345	0.8540
A4L	0.9125	0.0554	0.8185	1.0172	0.0985	0.0000	0.9275	2639	345	0.3370
A4S	0.9852	0.0555	0.8837	1.0984	0.7884	0.0000	0.6244	2639	345	0.8540
CA2	1.0718	0.0577	0.9572	1.2001	0.2295	0.0000	0.4751	2639	345	0.3851
CA3	0.9214	0.0578	0.8227	1.0318	0.1564	0.0000	0.5667	2639	345	0.3703
MHy	0.8179	0.0812	0.6976	0.9590	0.0133	0.4338	0.1838	2639	345	0.2922
MM	0.9439	0.0549	0.8477	1.0510	0.2925	0.0000	0.9701	2639	345	0.4388
TA2FS0	1.1037	0.0544	0.9920	1.2280	0.0698	0.0000	0.8238	2639	345	0.3370

Supplementary Table 11. Meta analysed associations between derived glycan traits and retinopathy in type 2 diabetes in the full model. Glycan values are centered and scaled. The *p*-values after FDR correction that are significant (≤0.05) are highlighted in green. Red represents a positive association and blue a negative association. Full model: age, sex, the interaction thereof, BMI, HDL, non-HDL, duration of diabetes, eGFR and HbA1c.

Prevalent (cross-sectional)										
**glycome**	**OR**	**SE**	**CIlower**	**CIupper**	**pval**	**I2**	**I2pval**	**n**	**nevent**	**pval.FDR**
A2E	0.9302	0.0525	0.8394	1.0310	0.1680	0.0000	0.7128	3217	505	0.7789
A2E0F	0.9831	0.0541	0.8842	1.0930	0.7521	0.0000	0.8989	3217	505	0.8461
A2EF	1.0539	0.0683	0.9218	1.2049	0.4421	0.3956	0.1983	3217	505	0.7789
A2F	1.0620	0.0522	0.9586	1.1764	0.2497	0.0000	0.3984	3217	505	0.7789
A2F0G	0.9290	0.0818	0.7914	1.0905	0.3677	0.6031	0.1125	3217	505	0.7789
A2F0GE	0.8988	0.0545	0.8078	1.0000	0.0501	0.0000	0.4366	3217	505	0.7789
A2F0GL	1.0315	0.0545	0.9269	1.1478	0.5700	0.0000	0.6011	3217	505	0.7843
A2F0GS	0.8838	0.0700	0.7705	1.0137	0.0774	0.3987	0.1972	3217	505	0.7789
A2F0S0B	0.9810	0.0540	0.8825	1.0906	0.7232	0.0000	0.9748	3217	505	0.8345
A2F0S0G	1.0795	0.0548	0.9696	1.2018	0.1627	0.0000	0.7094	3217	505	0.7789
A2F0SB	1.0494	0.0639	0.9259	1.1894	0.4500	0.3567	0.2125	3217	505	0.7789
A2FG	0.9694	0.0546	0.8710	1.0789	0.5687	0.0000	0.4771	3217	505	0.7843
A2FGE	1.0529	0.0555	0.9445	1.1738	0.3526	0.0000	0.6443	3217	505	0.7789
A2FGL	0.9634	0.0568	0.8619	1.0769	0.5115	0.0000	0.4428	3217	505	0.7843
A2FGS	1.0345	0.0551	0.9286	1.1524	0.5380	0.0000	0.9830	3217	505	0.7843
A2FS0B	1.0037	0.0574	0.8969	1.1233	0.9486	0.0000	0.9514	3217	505	0.9702
A2FS0G	0.9300	0.0610	0.8252	1.0481	0.2341	0.0000	0.6695	3217	505	0.7789
A2FSB	1.0619	0.0540	0.9553	1.1803	0.2660	0.0000	0.3614	3217	505	0.7789
A2FSG	0.9746	0.0548	0.8754	1.0850	0.6385	0.0000	0.4265	3217	505	0.7962
A2GS	0.9392	0.0532	0.8463	1.0424	0.2383	0.0000	0.4736	3217	505	0.7789
A2L	1.0347	0.0552	0.9286	1.1529	0.5364	0.0000	0.4396	3217	505	0.7843
A2L0F	1.0587	0.0525	0.9552	1.1734	0.2771	0.0000	0.3940	3217	505	0.7789
A2LF	1.0447	0.0551	0.9377	1.1639	0.4278	0.0000	0.4791	3217	505	0.7789
A2S0F	0.9896	0.0542	0.8899	1.1005	0.8472	0.0000	0.8362	3217	505	0.9077
A2SG	0.9191	0.0963	0.7609	1.1101	0.3811	0.7079	0.0643	3217	505	0.7789
A3E	0.9098	0.0643	0.8021	1.0319	0.1412	0.2111	0.2602	3217	505	0.7789
A3F	1.0545	0.0558	0.9453	1.1764	0.3413	0.0000	0.5354	3217	505	0.7789
A3L	1.0012	0.0560	0.8971	1.1174	0.9828	0.0000	0.5164	3217	505	0.9828
A3L0F	1.0975	0.0836	0.9316	1.2930	0.2659	0.5760	0.1246	3217	505	0.7789
A3LF	1.0479	0.0559	0.9392	1.1692	0.4022	0.0000	0.5170	3217	505	0.7789
A3S	0.9120	0.1092	0.7363	1.1296	0.3987	0.7608	0.0409	3217	505	0.7789
A4E	1.0224	0.0554	0.9172	1.1397	0.6896	0.0000	0.8578	3217	505	0.8166
A4F	1.0723	0.0560	0.9609	1.1967	0.2122	0.0000	0.7726	3217	505	0.7789
A4F0GE	1.0312	0.0548	0.9262	1.1480	0.5751	0.0000	0.6651	3217	505	0.7843
A4F0GL	1.0078	0.0552	0.9045	1.1230	0.8879	0.0000	0.6366	3217	505	0.9291
A4FGE	0.9834	0.0629	0.8693	1.1125	0.7902	0.2319	0.2539	3217	505	0.8673
A4FGL	1.0540	0.0660	0.9261	1.1997	0.4256	0.3054	0.2302	3217	505	0.7789
A4FGS	1.0617	0.0542	0.9547	1.1807	0.2694	0.0000	0.6817	3217	505	0.7789
A4L	1.0267	0.0557	0.9205	1.1452	0.6365	0.0000	0.8876	3217	505	0.7962
A4S	1.0861	0.0541	0.9768	1.2076	0.1269	0.0000	0.8530	3217	505	0.7789
CA2	1.0532	0.1132	0.8436	1.3148	0.6470	0.7544	0.0436	3217	505	0.7962
CA3	0.9426	0.1322	0.7274	1.2214	0.6547	0.8182	0.0190	3217	505	0.7962
MHy	0.8858	0.0750	0.7647	1.0260	0.1058	0.3736	0.2064	3217	505	0.7789
MM	0.9292	0.0532	0.8373	1.0313	0.1674	0.0000	0.9812	3217	505	0.7789
TA2FS0	1.0306	0.0518	0.9311	1.1408	0.5607	0.0000	0.8646	3217	505	0.7843

Supplementary Table 11. Meta analysed associations between derived glycan traits and retinopathy in type 2 diabetes in the full model. Glycan values are centered and scaled. The *p*-values after FDR correction that are significant (≤0.05) are highlighted in green. Red represents a positive association and blue a negative association. Full model: age, sex, the interaction thereof, BMI, HDL, non-HDL, duration of diabetes, eGFR and HbA1c.

Incident (Cox)										
**glycome**	**HR**	**SE**	**CIlower**	**CIupper**	**pval**	**I2**	**I2pval**	**n**	**nevent**	**pval.FDR**
A2E	0.9114	0.0550	0.8182	1.0152	0.0919	0.0000	0.7479	2639	345	0.6181
A2E0F	1.0434	0.0775	0.8963	1.2147	0.5836	0.4134	0.1917	2639	345	0.9043
A2EF	1.0412	0.0791	0.8916	1.2159	0.6097	0.4844	0.1637	2639	345	0.9043
A2F	1.0776	0.0558	0.9660	1.2022	0.1801	0.0000	0.3221	2639	345	0.6539
A2F0G	0.9082	0.1062	0.7375	1.1184	0.3648	0.7241	0.0569	2639	345	0.7461
A2F0GE	0.9666	0.0565	0.8652	1.0798	0.5478	0.0100	0.3149	2639	345	0.9043
A2F0GL	1.0080	0.0563	0.9027	1.1255	0.8880	0.0000	0.5391	2639	345	0.9747
A2F0GS	0.9664	0.0839	0.8199	1.1390	0.6832	0.5107	0.1528	2639	345	0.9043
A2F0S0B	1.0458	0.0546	0.9395	1.1640	0.4129	0.0000	0.7791	2639	345	0.7833
A2F0S0G	0.9202	0.0537	0.8283	1.0223	0.1213	0.0000	0.6996	2639	345	0.6181
A2F0SB	1.1201	0.0552	1.0052	1.2481	0.0400	0.0000	0.3668	2639	345	0.3600
A2FG	0.9147	0.0562	0.8193	1.0213	0.1128	0.0000	0.8626	2639	345	0.6181
A2FGE	0.9971	0.0983	0.8224	1.2089	0.9761	0.6341	0.0983	2639	345	0.9761
A2FGL	0.9952	0.0589	0.8867	1.1168	0.9343	0.0000	0.6504	2639	345	0.9761
A2FGS	1.0036	0.0786	0.8603	1.1707	0.9639	0.4304	0.1852	2639	345	0.9761
A2FS0B	1.0304	0.0592	0.9175	1.1571	0.6133	0.0000	0.7577	2639	345	0.9043
A2FS0G	0.9049	0.1016	0.7415	1.1044	0.3256	0.5766	0.1244	2639	345	0.7326
A2FSB	1.0462	0.0557	0.9379	1.1669	0.4178	0.0000	0.8300	2639	345	0.7833
A2FSG	0.9379	0.0580	0.8372	1.0508	0.2692	0.0000	0.7685	2639	345	0.6880
A2GS	0.9440	0.0622	0.8356	1.0665	0.3548	0.1595	0.2754	2639	345	0.7461
A2L	0.9947	0.0576	0.8885	1.1136	0.9267	0.0000	0.4600	2639	345	0.9761
A2L0F	1.0760	0.0557	0.9646	1.2001	0.1889	0.0000	0.3439	2639	345	0.6539
A2LF	1.0286	0.0584	0.9174	1.1532	0.6290	0.0000	0.8498	2639	345	0.9043
A2S0F	1.0278	0.0828	0.8738	1.2090	0.7404	0.4908	0.1611	2639	345	0.9157
A2SG	0.8885	0.0553	0.7972	0.9903	0.0327	0.0000	0.7624	2639	345	0.3600
A3E	0.9622	0.0744	0.8317	1.1133	0.6048	0.3803	0.2040	2639	345	0.9043
A3F	1.0652	0.0587	0.9494	1.1950	0.2822	0.0000	0.8101	2639	345	0.6880
A3L	0.9904	0.0574	0.8849	1.1084	0.8660	0.0000	0.6294	2639	345	0.9742
A3L0F	1.0881	0.0596	0.9681	1.2230	0.1566	0.0000	0.4101	2639	345	0.6408
A3LF	1.0633	0.0581	0.9489	1.1915	0.2905	0.0000	0.6347	2639	345	0.6880
A3S	0.9382	0.0558	0.8410	1.0467	0.2533	0.0000	0.3767	2639	345	0.6880
A4E	1.0183	0.0576	0.9096	1.1401	0.7527	0.0000	0.6090	2639	345	0.9157
A4F	1.0765	0.0587	0.9595	1.2077	0.2092	0.0000	0.7051	2639	345	0.6723
A4F0GE	1.0142	0.0572	0.9065	1.1346	0.8058	0.0000	0.8920	2639	345	0.9297
A4F0GL	1.0152	0.0569	0.9081	1.1349	0.7911	0.0000	0.8765	2639	345	0.9297
A4FGE	1.0191	0.0600	0.9060	1.1462	0.7529	0.1292	0.2839	2639	345	0.9157
A4FGL	1.0248	0.0568	0.9168	1.1455	0.6665	0.0000	0.7394	2639	345	0.9043
A4FGS	1.0931	0.0578	0.9760	1.2242	0.1236	0.0000	0.3755	2639	345	0.6181
A4L	1.0240	0.0578	0.9143	1.1469	0.6816	0.0000	0.7460	2639	345	0.9043
A4S	1.0848	0.0572	0.9698	1.2135	0.1545	0.0000	0.6862	2639	345	0.6408
CA2	1.1600	0.0610	1.0292	1.3074	0.0151	0.0000	0.7857	2639	345	0.3030
CA3	0.8435	0.0611	0.7483	0.9509	0.0054	0.0000	0.8280	2639	345	0.2422
MHy	0.9132	0.0749	0.7885	1.0575	0.2250	0.3251	0.2235	2639	345	0.6751
MM	0.9715	0.0563	0.8700	1.0850	0.6083	0.0000	0.8637	2639	345	0.9043
TA2FS0	1.1351	0.0546	1.0200	1.2632	0.0202	0.0000	0.9007	2639	345	0.3030
