# Supplementary material for: Plasma protein N-glycosylation is associated with cardiovascular disease, nephropathy, and retinopathy in type 2 diabetes
Source: BMJ Open Diabetes Res Care. 2021 Oct 13;9(1):e002345. doi: 10.1136/bmjdrc-2021-002345 (PMC8515459; doi:10.1136/bmjdrc-2021-002345)
Supplement: Supplementary data [file bmjdrc-2021-002345supp001.pdf]

The following material is supplementary to the manuscript:

Plasma protein *N*-glycosylation is associated with cardiovascular disease, nephropathy, and retinopathy in Type 2 diabetes.

Elham Memarian [a,b] *, Leen M 't Hart [c,d,e] *, Roderick C. Slieker [c,d], Roosmarijn F.L. Lemmers [f], Amber A van der Heijden [g], Femke Rutters [h], Giel Nijpels [g], Emma Schoep [c], Aloysius G. Lieverse [i], Eric J.G. Sijbrands [f], Manfred Wuhrer [a], Mandy van Hoek [f] **, Viktoria Dotz [a] **,#

[a] Center for Proteomics and Metabolomics, Leiden University Medical Center, Leiden, the Netherlands

[b] Genos Glycoscience Research Laboratory, Zagreb, Croatia

[c] Department of Cell and Chemical Biology, Leiden University Medical Center, Leiden, the Netherlands

[d] Department of Epidemiology and Biostatistics, Amsterdam University Medical Center, location VUmc, Amsterdam, The Netherlands

[e] Department of Biomedical Data Sciences, Section Molecular Epidemiology, Leiden University Medical Center, Leiden, the Netherlands

[f] Department of Internal Medicine, Erasmus University Medical Center, Rotterdam, the Netherlands

[g] Amsterdam UMC, Vrije Universiteit Amsterdam, Department of General Practice Medicine, Amsterdam Public Health Research Institute, Amsterdam, the Netherlands

[h] Amsterdam UMC, Vrije Universiteit Amsterdam, Department of Epidemiology and Data Science, Amsterdam Public Health Research Institute, Amsterdam, the Netherlands

[i] Department of Internal Medicine, Maxima Medical Center, Eindhoven, the Netherlands

*These authors contributed equally

**These authors contributed equally

# Current address: BioTherapeutics Analytical Development, Janssen Biologics BV, Einsteinweg 101, 2333 CB, Leiden, The Netherlands

Corresponding author:

Mandy van Hoek, ORCIDID: 0000-0002-2957-5436, Department of Internal Medicine, Erasmus MC – University Medical Center Rotterdam, 3015 GD, Rotterdam, The Netherlands, +31 633343603, m.vanhoek@erasmusmc.nl

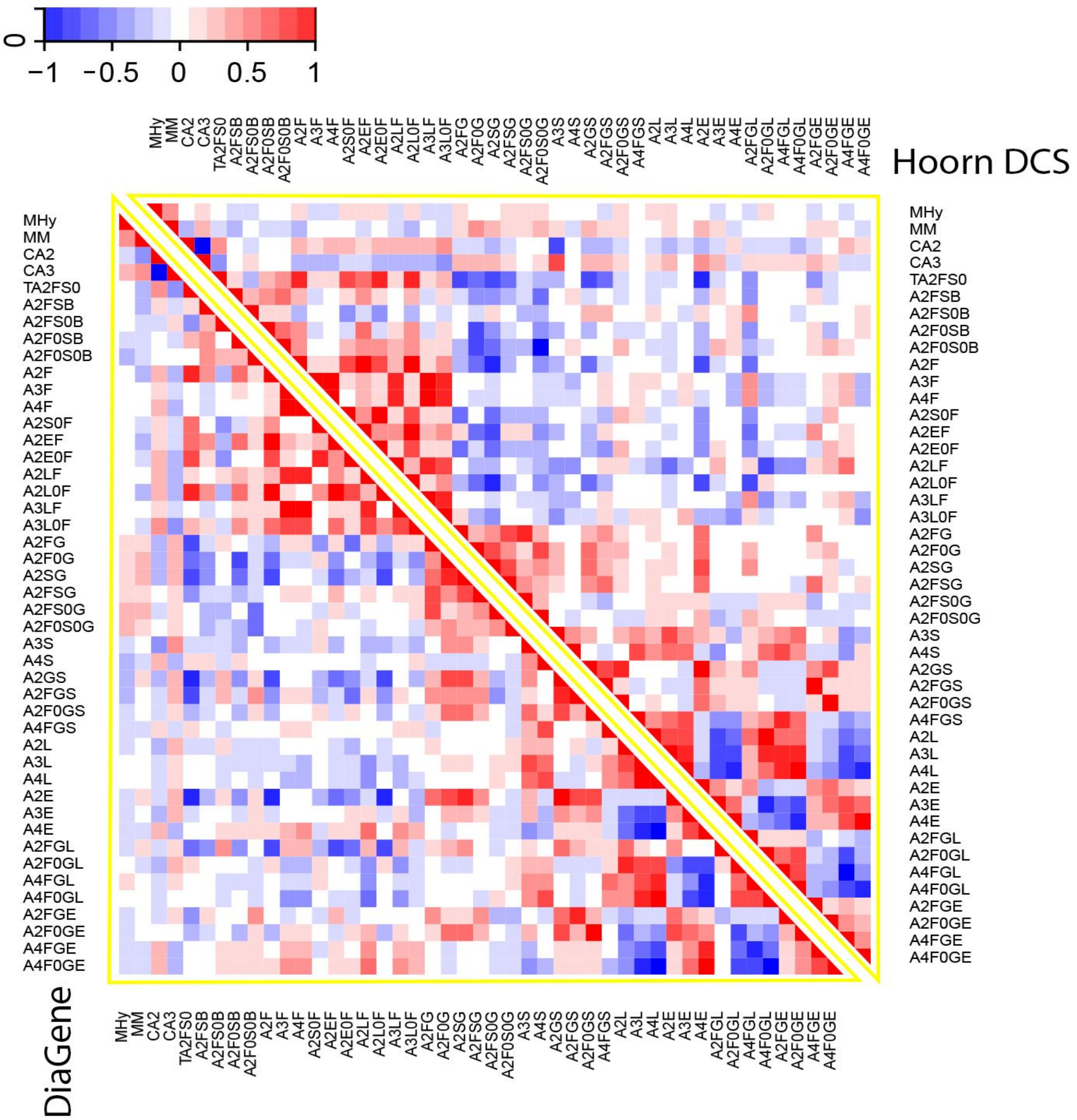

Supplementary figure 1: Heatmaps displaying the correlations between *N*-glycan derived traits in both cohorts, DiaGene and Hoorn DCS. Heatmaps were generated in R using the “heatmap.2” package. Sorting of the derived glycan traits in the heatmap was performed based on general glycosylation features, i.e., complexity, bisection (B), fucosylation (F), galactosylation (G), sialylation (total (S), α2,3-linked (L), and α2,6-linked (E)).

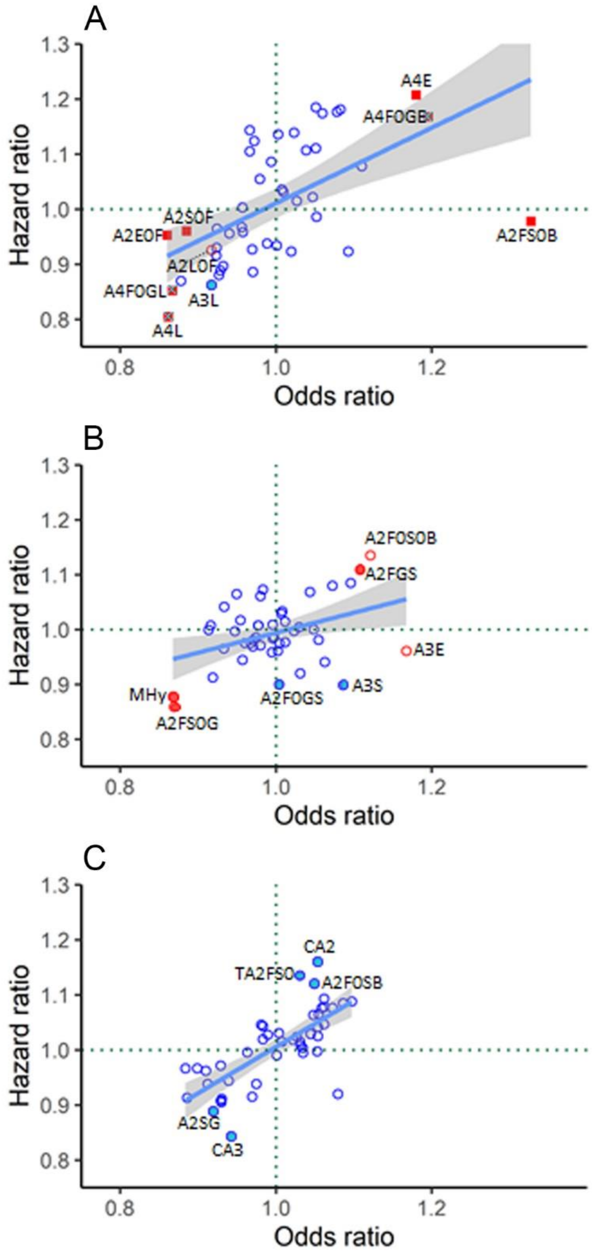

Supplementary figure 2: *N*-glycan derived traits hazard ratios plotted vs. odds ratios for meta-analysed data from DiaGene and Hoorn DCS studies in full model (adjusted for age, sex, the interaction thereof, BMI, HDL, non-HDL, duration of diabetes, eGFR and HbA1c).

(A) Cardiovascular disease (CVD), (B) Nephropathy, (C) Retinopathy. Red-filled square with blue cross: Significant in prevalent after FDR correction and in incident before FDR correction. Red-filled square: Significant in prevalent after FDR correction. Red-filled circle: Significant in prevalent and incident before FDR correction. Blue-filled circle: Significant in incident before FDR correction. Red unfilled circle: Significant in prevalent before FDR correction. Blue unfilled circle: Non-significant.
